# Fitting of Soil-Water Characteristic Curves (SWCC) of Bukit Mewah, Malaysia soil using field monitoring dataset

**DOI:** 10.1371/journal.pone.0316488

**Published:** 2025-01-10

**Authors:** Faris Shazani Suhaizan, Aizat Mohd Taib, Mohd Raihan Taha, Dayang Zulaika Abang Hasbollah, Aniza Ibrahim, Mohd Firdaus Md. Dan, Alfrendo Satyanaga

**Affiliations:** 1 Department of Civil Engineering, Faculty of Engineering & Built Environment, Universiti Kebangsaan Malaysia, Selangor, Malaysia; 2 Megaconsult Sdn. Bhd. Pusat Bandar Wangsa Maju, Kuala Lumpur, Malaysia; 3 Centre of Tropical Geoengineering, Faculty of Civil Engineering, Universiti Teknologi Malaysia, UTM, Johor Bahru, Malaysia; 4 Department of Civil Engineering, Faculty of Engineering, Universiti Pertahanan Nasional Malaysia, Kuala Lumpur, Malaysia; 5 Sustainable Geostructure and Underground Exploration, Faculty of Civil Engineering and Built Environment, Universiti Tun Hussein Onn Malaysia, Batu Pahat, Johor, Malaysia; 6 Department of Civil and Environmental Engineering, Nazarbayev University, Nur-Sultan, Kazakhstan; Ardakan University, ISLAMIC REPUBLIC OF IRAN

## Abstract

Rainfall-induced landslides are a frequent geohazard for tropical regions with prevalent residual soils and year-round rainy seasons. The water infiltration into unsaturated soil can be analyzed using the soil-water characteristic curve (SWCC) and permeability function which can be used to monitor and predict incoming landslides, showing the necessity of selecting the appropriate model parameter while fitting the SWCC model. This paper presents a set of data from six different sections of the studied slope at varying depths that are used to test the performance of three SWCC models, the van Genuchten-Mualem (vG-M), Fredlund-Xing (F-X) and Gardner (G). The dataset is obtained from field monitoring of the studied slope, over a duration of 6 months. The study discovered that the van Genuchten-Mualem model provided the best estimation based on RMSE and evaluation metric, R2 followed by Fredlund and Xing, and Gardner, however, the difference between them is minor. The R2 obtained varies as the value at the crest with 1.0 m depth has a mean of 0.44, the lowest among the overall data fitted but it also has the best RMSE value with a mean of 0.00473. Whereas the location mid-section at a depth of 1.0 m has the highest R2 with a mean of 0.97, and an average value of RMSE of 0.0145 which is the middle of the group that was fitted. This indicates that R2 measurement for model performance relies highly on the dispersion of the variables collected. The dispersion of the data set is mainly due to the sensors’ inability to detect effectively at exceedingly high matric suction and zero matric suction. The investment in improving the equipment’s precision will boost reliability and reduce the number of assumptions as the data is collected from the site rather than laboratory testing.

## Introduction

In Malaysia, residual granite rock soil and sedimentary rock soil occur extensively, i.e. cover more than 80% of the land area [1] and more than 70% of the land area in Malaysia is covered by residual soil [2]. In the tropical region of Southeast Asia, Malaysia experiences high rainfall virtually all year long, but especially from October to February [3]. The groundwater table is generally low causing the soil to be mostly unsaturated except immediately after rainfall which decreases the negative pore-water pressure. As a result, the soil’s shear strength is reduced, which increases the slope’s risk of failing [4].

Heavy rainfall tends to induce the majority of shallow slope failures/landslides [5–7], especially during the rainy season, resulting in property damage, and even causing injury and fatality [8, 9]. Rainfall-induced landslides, which are among the most expensive and dangerous natural disasters [10] and are also a frequent geohazard for tropical and subtropical regions where residual soils are prevalent, are ubiquitous in hillslope situations all over the world [11–14]. This concern was further amplified as the slopes tend to fail even after a rainfall event occurs thus the need to further the understanding of the unsaturated state of soil is needed in depth.

Numerous authors cited the rapid rise in pore water pressure and the establishment of positive pore pressures brought on by the formation of a perched water table as the primary factor in the occurrence of shallow landslides [15–19]. The water infiltration also known as seepage, causes soil that is initially in an unsaturated condition to lose a considerable amount of its shear strength, which makes the slope unstable [20, 21]. The seepage of unsaturated soil is governed by the suction variations and permeability function, both of which are the two main factors governing the soil-water characteristic curve (SWCC) [22]. In vadose zone hydrology and contemporary soil mechanics, the SWCC is regarded as a crucial constitutive function [23–27]. Thus, it is important to understand and grasp the SWCC to avoid and predict future slope failure.

The conventional method for measuring characteristic curves (SWCC) is through the utilization of the pressure plate method. However, this approach is associated with high costs, significant labor requirements, and time consumption. In laboratory settings, SWCC tests are often conducted without confining pressure, leading to potential inaccuracies in the acquired data, as noted by Satyanaga et al. [28]. Obtaining actual SWCC measurements in the field is the most optimal approach, as it considers the impact of prevailing climatic conditions on the scanning curve of SWCC.

In addition to gathering information on appropriate model parameters for the site soil, the objective of this study is to assess the effectiveness of the novel suction probe and water content sensors for measuring the residual soil’s soil-water characteristic curve in real time. Field monitoring data collecting for water content and suction, as well as climate data monitoring within a Bukit Mewah slope, are all included in the scope of work. The best mathematical models for Bukit Mewah’s soil slope would be compared using performance evaluation and the soil water characteristic curves from field measurements. Thus, this paper’s new contribution is the model’s application to slope soils through careful soil suction and water content monitoring.

## Background and theoretic

### soil-water characteristic curve

For tropical climate regions, the intense rainfall and harsh sunlight come hand in hand with the geography. The soil slope will undergo multiple cycles of permeability from the precipitation and evaporation from the sunlight. To understand this relationship and its effect on the shear strength of the soil, unsaturated soil mechanics is valuable knowledge. The interrelation of the soil and the water which was first termed by Buckingham [29] as the soil-water characteristic curve (SWCC) can also be used to describe the water movement in the soil [30, 31], the soil’s permeability [32, 33] and the shear strength of the soil [16, 34]. SWCC is a relationship between the moisture content and the negative pore water pressure which is suction in the soil.

**Fig 1:**
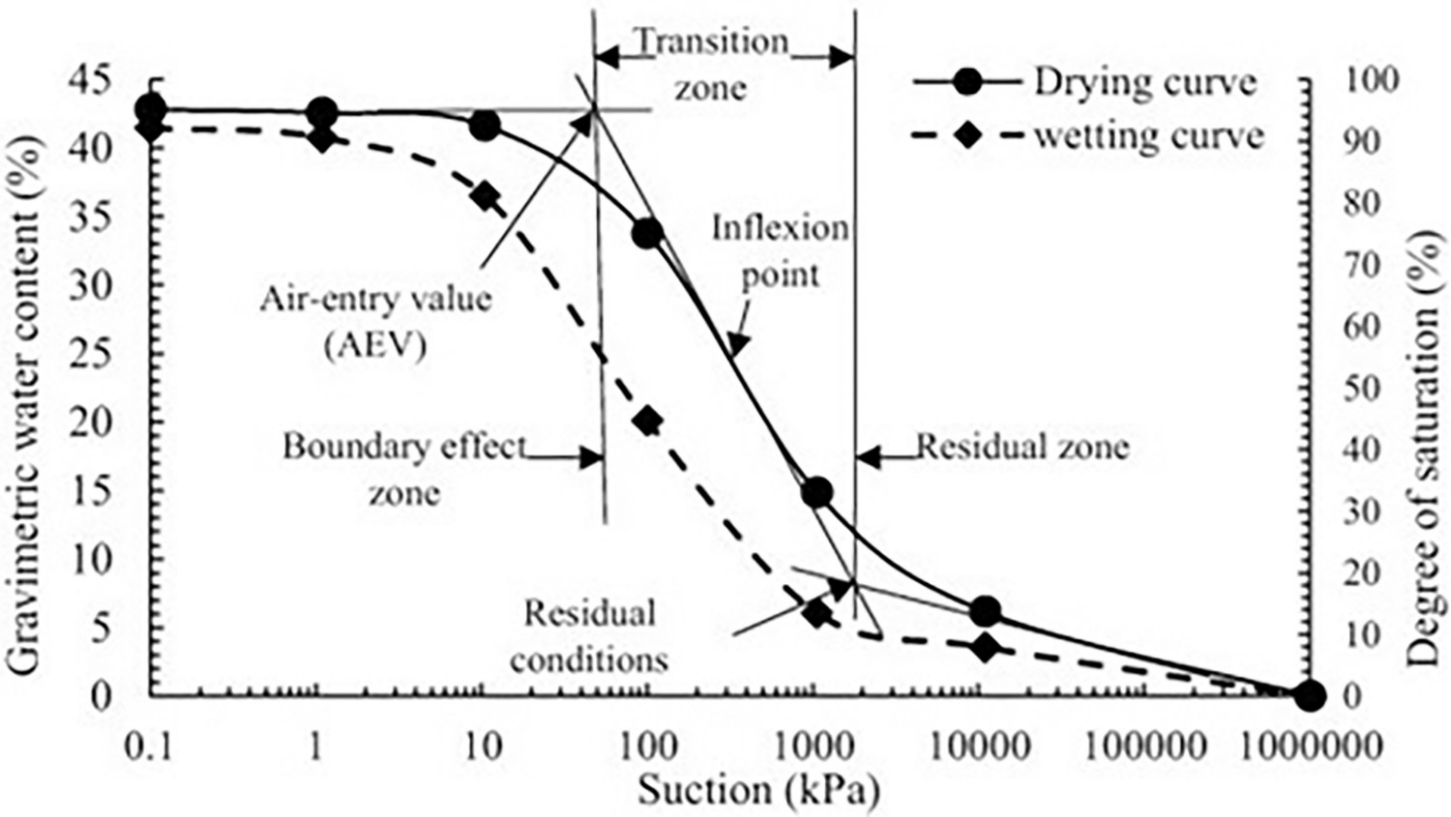
SWCC as illustrated by Eyo et al [46].

The typical SWCC is shown in **Fig 1** with the water content represented on the y-axis and the matric suction on the x-axis. The SWCC is a sigmoidal curve that is divided into three zones which are the boundary effect zone, transition zone, and residual zone. The curve also consists of an air entry value (AEV) where the voids of the saturated soils are entered by air due to suction. The phenomenon will continue and slowly reach the point where the water moisture is unable to exit the soil based on suction alone, this is called the residual zone.

### Field monitoring

Slope stability is an important part of geotechnical engineering, and field monitoring is crucial in assessing and managing potential risks related to slope instability. Field monitoring methods aim to provide real-time data on slope behavior, facilitating early detection of potential failures and guiding the development of risk mitigation techniques [35, 36]. For monitoring, researchers and engineers generally concentrate on pore pressure, hydrological conditions, and slope deformation both above and below the soil surface when discussing slope stability [15, 37–41].

It has long been known that matric suction plays a critical role in the stability of unsaturated steep residual soil slopes at shallow depths [42]. Even though several field investigations and laboratory test results have been published in the literature, it is still unknown how variances in water content, matric suction, and groundwater table relate to rainfall infiltration. [40]. Multiple field investigations have been done to evaluate how rainwater infiltration affected slope stability using field monitoring [15, 37, 39–40]. Some studies used field monitoring to observe variations in water content affected by rainfall to develop an early warning system for slope and embankment failure [36, 41]. Research in rainfall infiltration and the effects of antecedent rainfall on matric suction of soil and the water content has also been done in long-term periods to obtain a wide and extensive range of data [38, 43, 44]

### Model parameter

In modeling the SWCC, parameters are used to represent a set of functions and variables that describe the relationship between soil-water content and matric suction. The most commonly used parameters in SWCC models are a, n, and m. The parameter a represents the suction at the inflection point of the SWCC curve, and its value is related to the air-entry value of the soil being modeled. This parameter can be expressed in terms of suction or the inverse of suction.

Parameter n is typically associated with the slope of the SWCC curve in the desaturation zone and is related to the soil’s pore size distribution. Both the a and n parameters have physical significance and can be validated through testing and recorded data. Some SWCC models may include a third parameter, m, which describes the asymmetry of the SWCC curve around the inflection point. This parameter is often used to improve the fit of the model to the soil being modeled. Overall, these fitting parameters and choosing an appropriate equation from among those accessible in the literature play a crucial role in accurately representing the SWCC of a particular soil and can aid in understanding soil behavior and water movement [45].

#### a) Van Genuchten (1980)

The Van Genuchten SWCC model [47] is a widely used empirical model for describing the relationship between soil-water content and matric suction. The model was first proposed by Van Genuchten in 1980 in his paper and has become one of the most popular models for characterizing SWCCs in various fields, including hydrology, soil science, and agriculture. The van Genuchten equation model is shown in Eq (1).

Numerous studies have demonstrated the effectiveness of the Van Genuchten model in predicting soil-water retention curves. For example, Haverkamp and Parlange [48] evaluated the Van Genuchten model’s ability to predict SWCCs in a variety of soils and found that it performed well across a broad range of soil types and textures. Similarly, Šimůnek et al. [49] used the model to successfully simulate water movement and solute transport in unsaturated soils. Leong and Rahardjo [50] investigated the applicability of the Van Genuchten equation for various soil types and found that the equation was suitable and provided an excellent fit to experimental data.

$$\theta (\psi )=\theta_{r}+\frac{\left(\theta_{s}-\theta_{r}\right)}{{\left[1+{\left(a\psi \right)}^{n}\right]}^{m}}$$ (1)

Where:

*θ =* calculated volumetric water content

*θ*_*s*_ = saturated volumetric water content

*θr* = residual volumetric water content

*ψ* = matric suction under consideration (kPa)

*α* = fitting parameter related to inverse value of air entry of soil (kPa)

*n* = fitting parameter related to the maximum slope of curvature

*m* = fitting parameter related to the curvature of the slope

The Van Genuchten equation can capture the smooth transition from saturated to unsaturated conditions and is particularly useful for characterizing soil-water retention properties. Researchers have made modifications to the Van Genuchten model to address specific soil characteristics and other factors. For example, Xing et al. [51] modified the SWCC model based on the soil capillary theory to incorporate soils mixed with additives.

Despite its widespread use and effectiveness, some researchers have noted limitations and potential improvements for the Van Genuchten model. For example, Šimůnek et al. [52] highlighted the need to better understand the physical significance of the model’s fitting parameters and to account for scale-dependent effects in model development. Overall, the Van Genuchten SWCC model remains a valuable tool for characterizing soil-water retention curves and has contributed significantly to our understanding of unsaturated flow in soils.

#### b) Fredlund and Xing (1994)

Among the models that are developed for the SWCC, Fredlund, and Xing (1994) equation [33] is considered to be one of the most popular SWCC equations. The curve defined by Fredlund and Xing’s equation is mainly controlled by the fitting parameters as illustrated in Eq (2). The four independent parameters pertaining to the air entrance value, pore size distribution, residual water content, and pore connectivity are included in the model, which uses the Brooks and Corey (1964) model [53] for estimating the hydraulic conductivity function.

The Fredlund and Xing model, which is frequently used in soil research and geotechnical engineering, has been used to predict the SWCC of several soil types, including sandy and clayey soils. The model’s performance has been assessed in numerous studies for a range of soil types and environmental factors. For instance, Shen et al. [54] establishes that the Fredlund-Xing model best fits the SWCC suitable for assessing soil-water characteristics of calcareous silty sand which is a special type of geomaterial that is created from the organic remains of hermatypic corals and other marine life. Aldaood [55] examined the Fredlund and Xing model on silty sand soil and discovered that the model offered an accurate fit to the measured SWCC even at different percentages of fine materials. Similarly, Gallage and Uchimura [56] found that the Fredlund and Xing model correctly predicted the drying and wetting path of SWCCs for four sandy soils with different dry densities.

In summary, it has been demonstrated that the Fredlund and Xing model can accurately estimate the SWCC of various soils. It is crucial to calibrate the model for certain soil types and environmental conditions because soil composition, mineralogy, and environmental conditions may have an impact on the model’s accuracy.

$$\theta (\psi )=\theta_{r}+(\theta_{s}-\theta_{r}){\left[\frac{1}{\ln\left[e+{\left(\frac{\psi }{a}\right)}^{n}\right]}\right]}^{m}$$ (2)

Where:

*E =* base of natural logarithm

*α* = fitting parameter related to value of air entry of soil (kPa)

*n* = fitting parameter related to the maximum slope of curvature

*m* = fitting parameter related to the curvature of the slope

#### c) Gardner (1958)

Gardner (1958) proposed an empirical model [31] for describing the soil-water characteristic curve (SWCC) of unsaturated soils. The model uses an equation with two fitted parameters to build a link between soil-water content and matric potential between four independent parameters to fit a wide field of soil types. The Gardner model has been acknowledged and widely used in various fields as a reputed soil-water characteristic curve model in soil science and engineering.

The effectiveness and application of the Gardner model in forecasting the SWCC for various soil types and environmental conditions have been investigated in several studies. For instance, Agus et al. [57] applied the Gardner model to tropical peat soils and found that it provided a good fit to the experimental data. Furthermore, the Gardner model has also been used by Ng’eno et al [58] to study the moisture retention properties of the three different soil types and Super absorbent polymer (SAP) treated soils and was proven successful in representing the soil-water retention curve (SWRC).

In addition to its wide application, the Gardner model has also been modified to account for specific soil characteristics and environmental factors. Li et al. [59] proposed an improved Gardner model that considered the impact of soil organic matter content on the SWCC of loess soils. The improved model demonstrated better accuracy in capturing the complex water retention behavior of organic-rich soils. The Gardner water retention model and Archie’s second rule were utilized by Liu et al. [60] to create a new relationship between suction and soil electrical resistivity. The model presents a novel possibility to investigate the coupled transport of water and solutes in the field and to assess in situ suction dynamics.

In general, the Gardner SWCC model has proven to be a valuable tool for predicting the water retention behavior of unsaturated soils. However, it is essential to calibrate the model parameters for specific soil types and conditions to ensure accurate predictions.

$$\theta (\psi )=\theta r+\frac{\left(\theta_{5}-\theta_{r}\right)}{\left[1+{\left(a\psi \right)}^{\eta }\right]}$$ (3)

Where:

*θ* = calculated volumetric water content

*θ*_*s*_ = saturated volumetric water content

*θ*_*r*_ = residual volumetric water content

*ψ* = matric suction under consideration (kPa)

*α* = fitting parameter related to inverse value of air entry of soil (kPa)

*n* = fitting parameter related to the maximum slope of curvature

### Study area

#### Location

**Fig 2:**
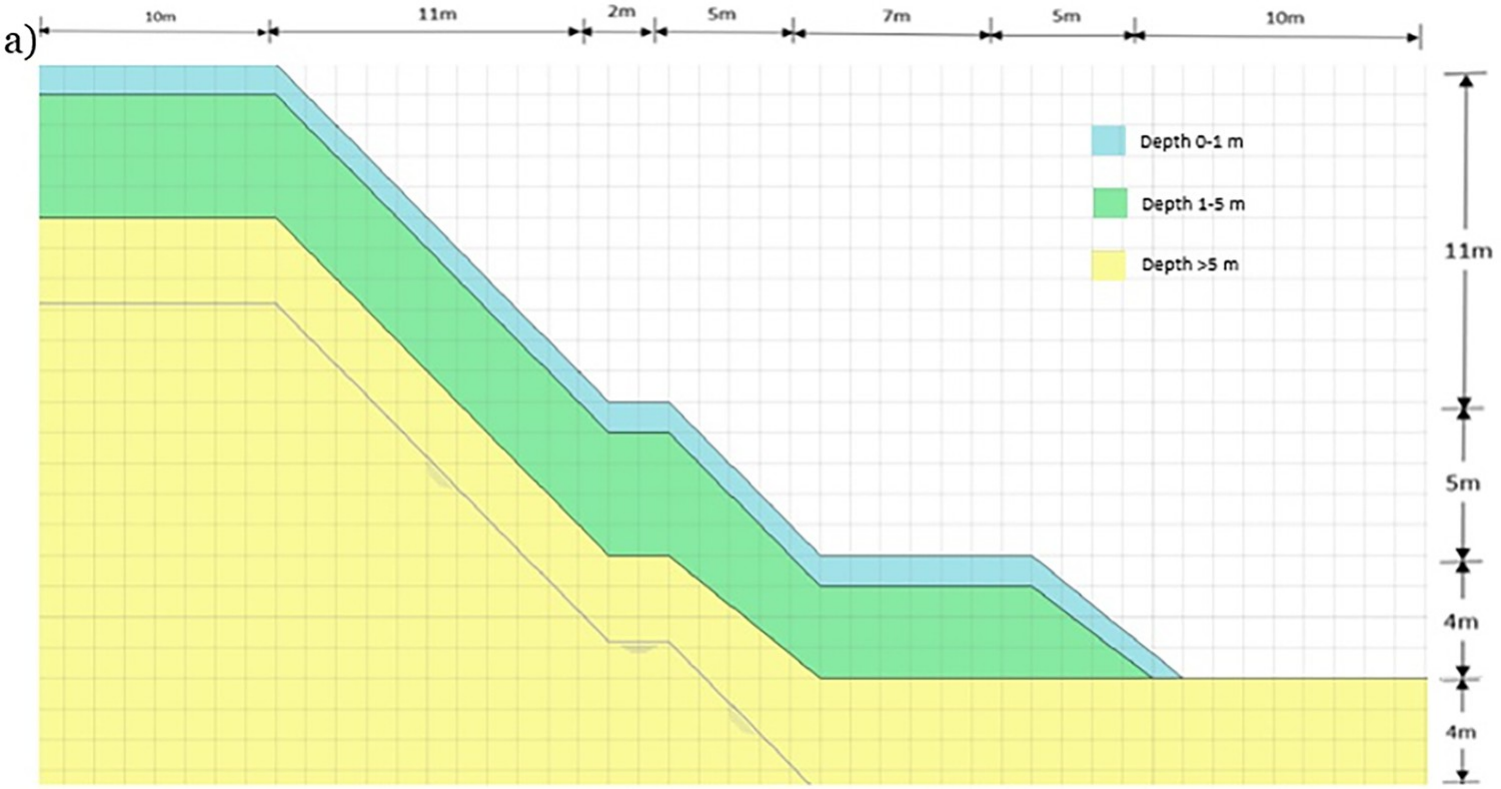
Slope geometry of slope Bukit Mewah.

The slope site is located geographically in Malaysia which is an equatorial tropical climate slightly above the equator line. The region has high intense rain and humidity while also having harsh sunlight throughout the year. The slope of the study site is in an area called Bukit Mewah, which is located in Kajang and the coordinates for the slope site are 2◦59’11" North and 101◦47’57" East. The crest of the slope is full of low-lying vegetation and is beside a residential road with houses built on the opposite side of the road. The location is considered a hilly area with residential buildings occupying most of the vicinity. The site’s slope is 45 degrees steep from the toe slope to the middle berm and from the middle berm to the crest of the slope as can be seen in Fig 2

**Fig 3:**
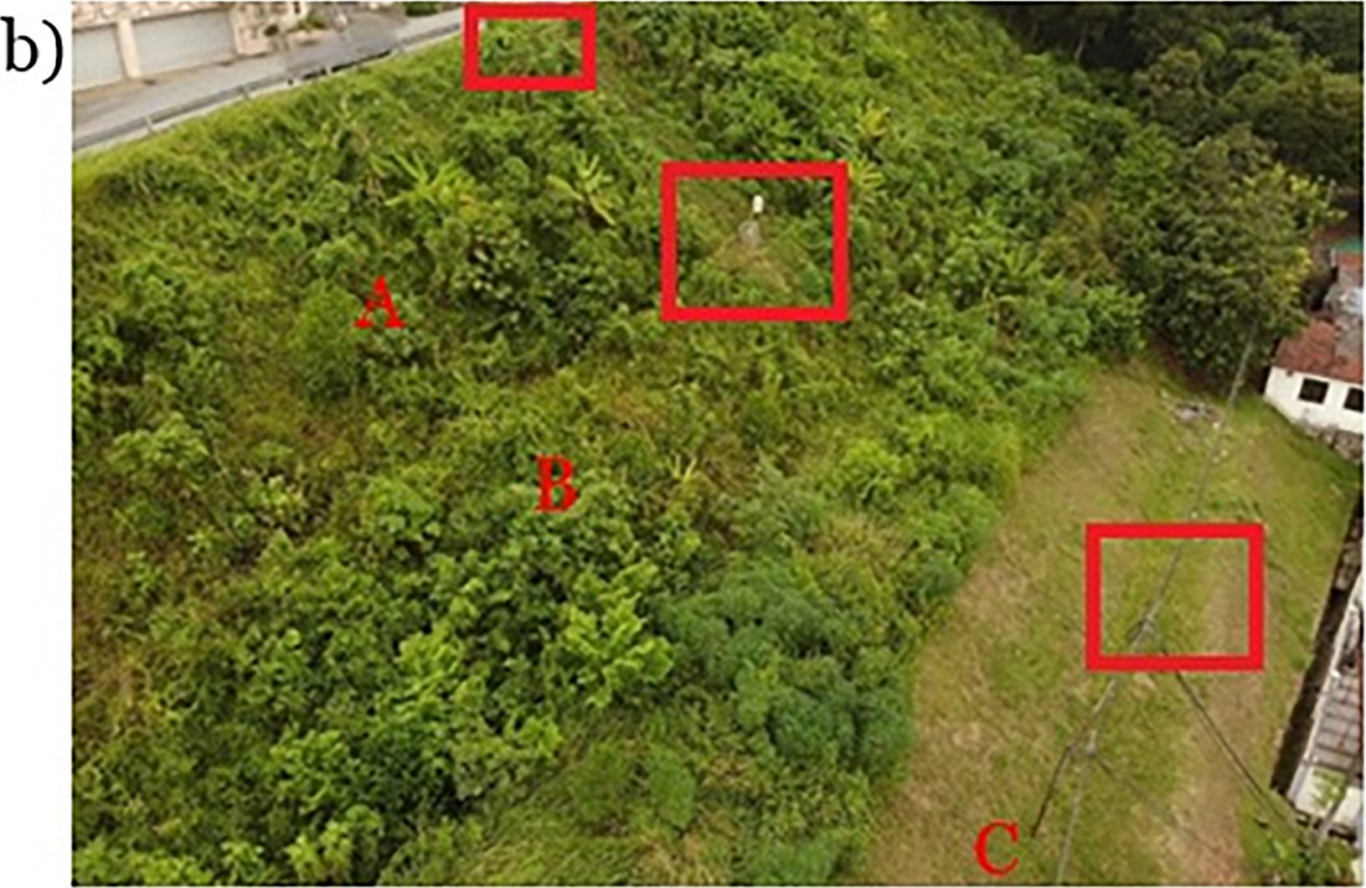
Landscape of studied slopes with sensors location.

**Fig 4:**
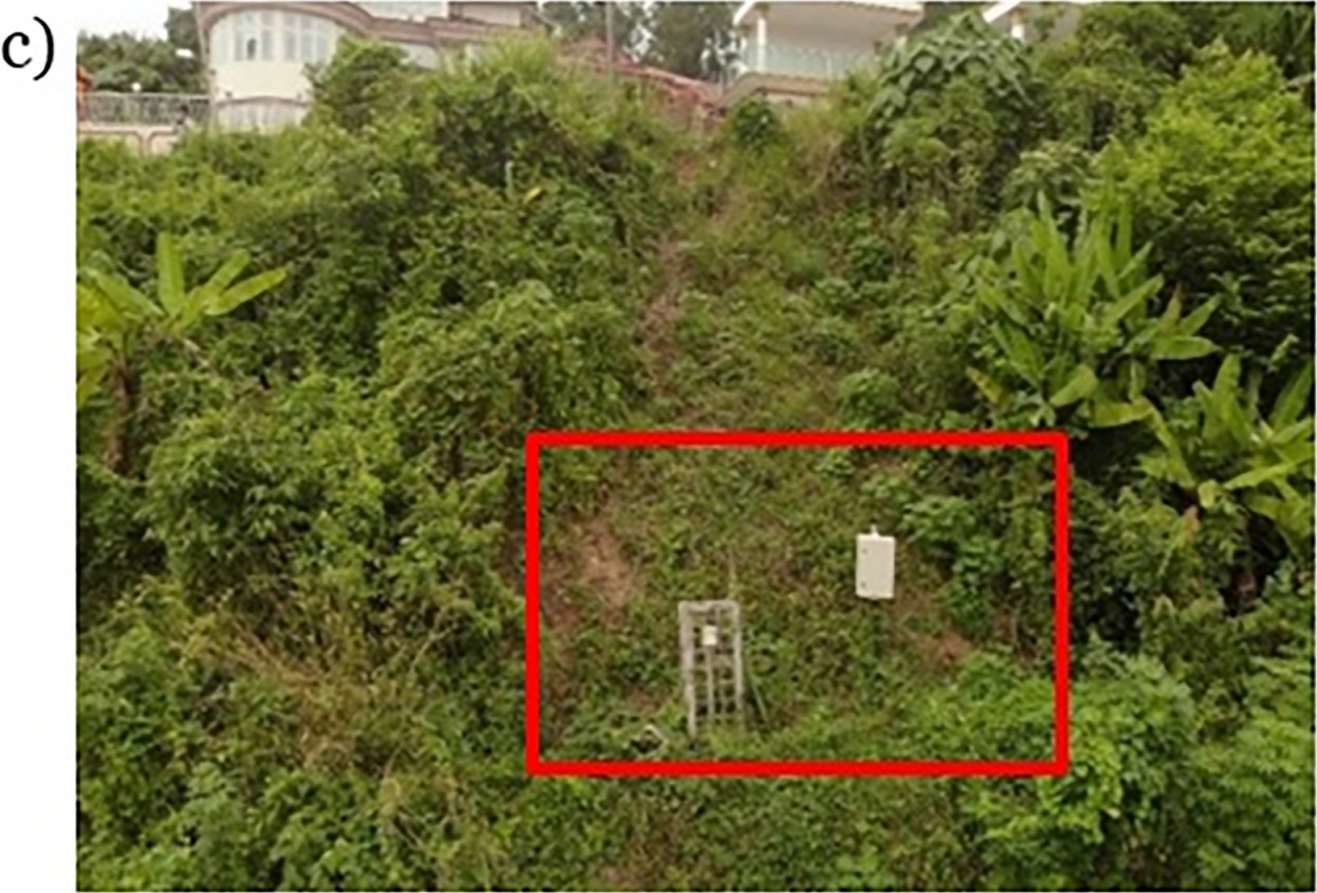
Field monitoring enclosure on site.

There are trees at the toe section of the slope and a residential housing area at the toe of the slope. There is no canopy or tall terrain in the vicinity of the field monitoring site except the slope itself is covered by a high density of low-lying vegetables, thus the face of the slope is exposed to the rainfall and sunlight without much blocking it accepts few foliage as seen in Figs 3 and 4.The access route to the slope is from the crest side of the slope beside the residential road.

For the studied slope, boreholes were drilled on the slope as part of the site investigation. The result of the investigation showed that the geological structure of the slope is close to homogeneous throughout with slight variances in depth. For the study done, the soil formation which is the main subject of this study is the top layer from topsoil at 0 m depth to 1 m. A simplified slope profile is presented in Table 1, which consists of three soil layers. The first layer is from the surface to 1m depth, the soil is soft to medium stiff and comprises medium brown silty clay indicating a moderate amount of organic matter which contributes to the soil’s dark coloration which also signifies good drainage and aeration. The coloration of medium brown color is often due to the presence of iron oxides and clay minerals.

**Table 1:** Slope profile.

Depth (m)	Stiffness	Coloration	Composition
0–1	Soft to medium stiff	Medium brown	Silty Clay
1–5	Medium stiff	Greyish brown	Silty Clay
>5	Stiff	Medium brown	Clay

The second layer stretches from 1 m to 5 m depth and is made of medium stiff, greyish brown silty clay. The brown coloration suggests the presence of some organic matter or oxidized iron, but the greyish tones often indicate poor drainage or anaerobic conditions and experience intermittent saturation, such as a seasonally wet area. The medium stiffness suggests that the soil is moderately compacted and has some load-bearing capacity. The last layer, which comprises more than 5 m of depth is made of stiff medium brown clay which is similar in color to the first layer. However, the stiffness implies that the soil has undergone some degree of drying or compression, possibly due to overburden pressure or prolonged exposure to load. The coloration observed in the soil is common in temperate climates where organic material breaks down at a moderate rate, balancing between accumulation and decomposition. The groundwater level was at a depth of 7.80 m from the borehole conducted at the crest of the slope.

## Methodology

### Soil investigation

The site had performed a site investigation to explore the subsoil conditions for the Bukit Mewah slope. The study was given permission by Kajang Municipal Council of the Engineering Department. The boring was done by using the rotary wash boring to obtain a good sample of undisturbed specimens that needed to be used for laboratory testing. All the samples were collected, checked by visual examination, and stored properly by a trained technician. There were multiple tests that have been done to obtain the properties of the slope. The tests were performed using the British Standard Methods of Test BS: 1377 [61] which is a widely recognized standard that provides detailed procedures for testing the properties of soil.

There were seven tests done for the two categories of Soil Index Properties and Soil Engineering Properties. For Soil Index Properties, four tests were done which are moisture content determination using oven drying method, grain size distribution using sieve and hydrometer analysis, density determination, and Atterberg limits. For the Soil Engineering Properties, three tests were done which are the triaxial CIU test, unconfined compressive strength test, and permeability by constant head method.

### Field monitoring

For the gathering of data for field monitoring, a Data Acquisition System for the systematic collection, analysis, and use of data was implemented during the project. An elementary data acquisition system consists of sensors, measurement hardware, and a computer with programmable software. The main computer, which is also known as a logger is situated inside an enclosure installed in the middle of the slope. The system implemented mainly has three main important data to record which are the negative pore pressure (Suction), volumetric water content, and precipitation.

The instrumentation chosen to monitor these parameters is chosen carefully according to the requirements of the research, condition, and layout of the slope sites. The suction measurement for the soil relies on TEROS 21 and the volumetric water content measurement is measured by TEROS 11. They are placed in pairs in 3 different locations which are the crest (A), mid-slope (B), and toe (C), and at two different depths for each location and they were installed horizontally at depths of 0.5 m and 1 m as seen in Figs 2 and 3. The data collected for 0.5 m and 1 m are for unsaturated behavior change that occurs during and after precipitation occurs.

The study site is also equipped with an ECRN-100 rain gauge to record the rainfall occurring on the site. METER Group from the USA is the manufacturer of the field measurement devices, TEROS 11, TEROS 21, and ECRN-100 rain gauge, which were produced in 2019. The data is stored on the logger and in a cloud system setup to ensure the data can be easily accessed and backed up if anything happens to the logger during the field monitoring period. This will provide a good all-encompassing data set to study the effect of unsaturated soil phenomenon occurring in the presence of rainfall.

### SWCC model

The unsaturated behavior of the data is represented by the statistical model developed by van Genuchten-Mualem variation (1980), Fredlund-Xing model (1994), and Gardner (1958). Utilizing these statistical models to determine the SWCC of the soil is appropriate because it has been thoroughly researched and acknowledged in the geotechnical industry.

The SWCC for the models is generally governed by 4 or 5 independent parameters that are mostly made up of the model parameters which are the *a*, *n*, and *m*, and the soil properties parameters such as the residual water content, saturated water content, parameter for the pore-size distribution and textural pore-space. In order to prevent incompatible fitting, the model parameters are constrained by empirical approaches to vary within a pre-specified range. For the models used for fitting in the study, the independent parameters are made of *a*, *n*, *m*, *θ*_*r*,_ and *θ*_*s*_.

The “*a*” parameter or the inverse of it for models such as van Genuchten-Mualem is confined to a range of 5 kPa to 10 kPa with an increment of two decimals places. The parameter “*n*” is restricted to a minimum of 1.0 and a maximum of 15.0 with an increment of one decimal place, while the value of “*m*” is limited to the range of 0.1 to 2 with an increment of two decimal places. These boundaries are applied to every model to guarantee that fitting does not rely on huge numbers to achieve a better fit as it may be a localized incidence and does not accurately reflect the overall soil structure under study.

## Result and discussion

### Soil properties

Soil properties are divided into two categories which are Soil Index Properties and Soil Engineering Properties. Both categories are needed for engineers and designers to check, monitor, and design a slope. In this thesis, both categories are needed to explore the nuances, inter-relation, and situation of unsaturated soil present in the site. For each different test, the samples are chosen by the solidity and uniformity of the sample obtained such as a more uniform and solid sample would be used for the CIU test, and the least uniform and solid would be used for the Soil Index Properties test. Both soil properties are tabulated in **Tables 2** and **3**.

**Table 2:** Soil index properties.

Soil Properties	Value
Moisture Content (%)	33
Specific Gravity	2.632
Void Ratio	0.65
Linear Shrinkage (%)	6
**Atterberg Limits**	
Liquid Limit (%)	36
Plastic Limit (%)	27
Plasticity Index (%)	9
**Particle Size Distribution**	
Clay (%)	5
Silt (%)	30
Sand (%)	21
Gravel (%)	44

**Table 3:** Soil engineering properties.

**Triaxial CIU**	
**Total Stress Parameters**	
Cohesion (kPa)	35
Angle of Shearing Resistance (Deg)	12
**Effective Stress Parameters**	
Cohesion (kPa)	5
Angle of Shearing Resistance (Deg)	29
**Permeability Test**	
cm/s	1.03E-07
**Unconfined Compression Test**	
kN/m^3^	237.3

**Fig 5:**
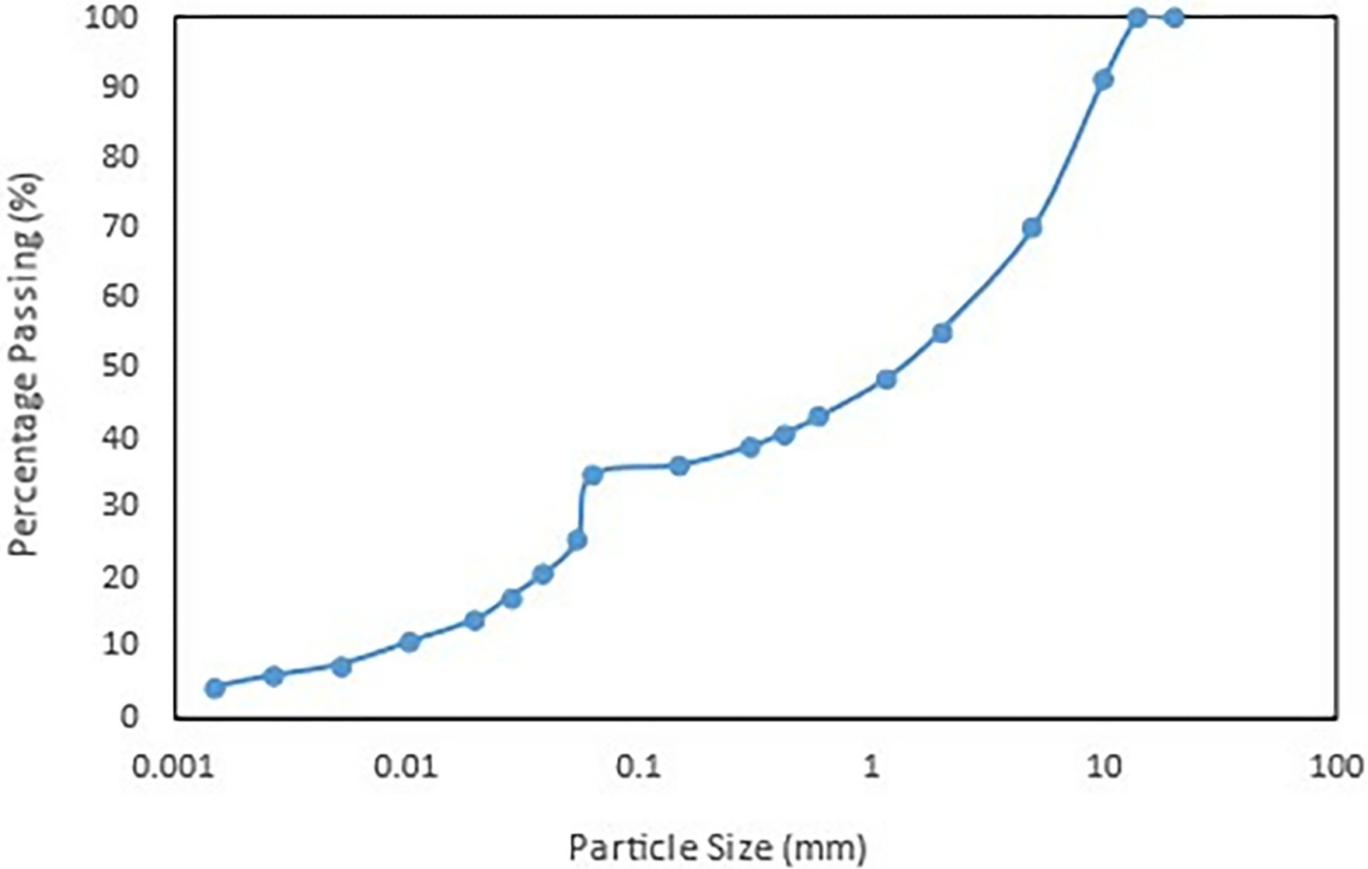
The particle size distribution of the soil slope.

Wet sieve analysis was also done to determine and classify the type of soil and the gradation and was charted in **Fig 5**. The sample’s moisture content, which measures how much water is in the soil in relation to its dry mass, was 33%. The liquid limit (LL) was 36%, the plastic limit (PL) was 27%, and the plasticity index (PI) was 9%, according to the Atterberg Limits, which are essential for determining the consistency and plasticity properties of the soil. The soil is less prone to experience notable volumetric changes in response to changes in moisture content, as indicated by the comparatively low flexibility index. Further characterising the soil’s shrink-swell potential is the linear shrinkage, which was measured at 6% and showed a moderate tendency for the soil to lose volume when dry.

According to the breakdown of particle sizes, the soil’s composition was as follows: 21% sand, 30% silt, 5% clay, and a significant 44% gravel. The Unified Soil Classification System (USCS) classifies the soil as silty gravel based on these proportions which is different that the actual field observation obtained from the boring log which is silty clay. This disparity between the field observations and the laboratory classification raises the possibility that the borehole has localised differences in the composition of the soil. Because soils can vary significantly in space, even across short distances, due to variations in depositional conditions, soil formation processes, or past geological activity, this phenomenon is not unusual.

### Field data records

**Fig 6:**
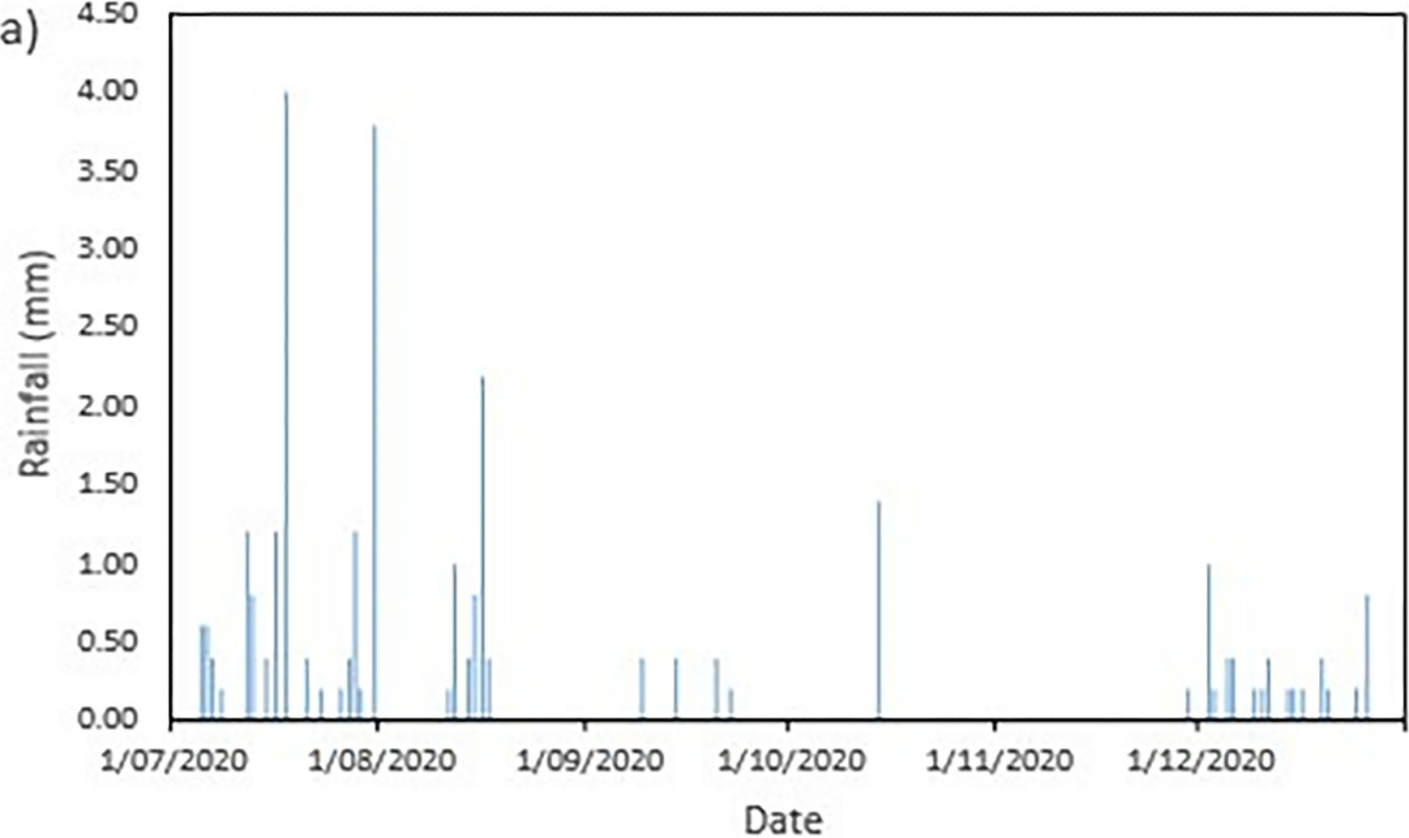
Precipitation records obtained from field monitoring.

The data collected from the field monitoring for a duration of 6 months from 1st July 2020 to 31st Dec 2020. **Fig 6** presents the meteorological data recorded on site; the data of the rainfall is slightly impaired due to the inability to do regular maintenance on the rain gauge as COVID lockdown occurred in Malaysia during the time. As a result, debris continues to collect at the opening of the funnel. This dampens the accessibility of the rainwater to be collected and recorded properly by the rain gauge. Recorded data collected from the sensors for both the volumetric water content and suction is presented in Figs **8–17** respectively.

The data is presented in the form of 5 figures each which represent the readings at the depth of 1 m and 0.5 m which are **Figs 8 and 9** for water content and **Figs 13 and 14** for suction. The representation of the sensors data for each location with depth are also shown for the crest, mid-slope and toe which are **Figs 10–12** for water content and **Figs 15–17** for suction. The readings are collected concurrently with the rainfall data which is during the 6 months period of the field monitoring. There are some readings that were lost due to the equipment malfunctioning that occurred during the recording period. The sensors are buried at a shallow depth from the surface to collect unsaturated behavior represented in the soil. A summary of data used is provided in S1 File.

#### a) Rainfall vs time graph

Based on the collected findings in Fig 6, it can be seen the highest amount of precipitation in a day was a total of 4 mm on 18 July 2020 which is followed by 3.8 mm on 31 July 2020. The highest amount of rainfall event was recorded during the month of July with a total of 16 rainfall events which has a total amount of precipitation of 15.8 mm.

**Fig 7:**
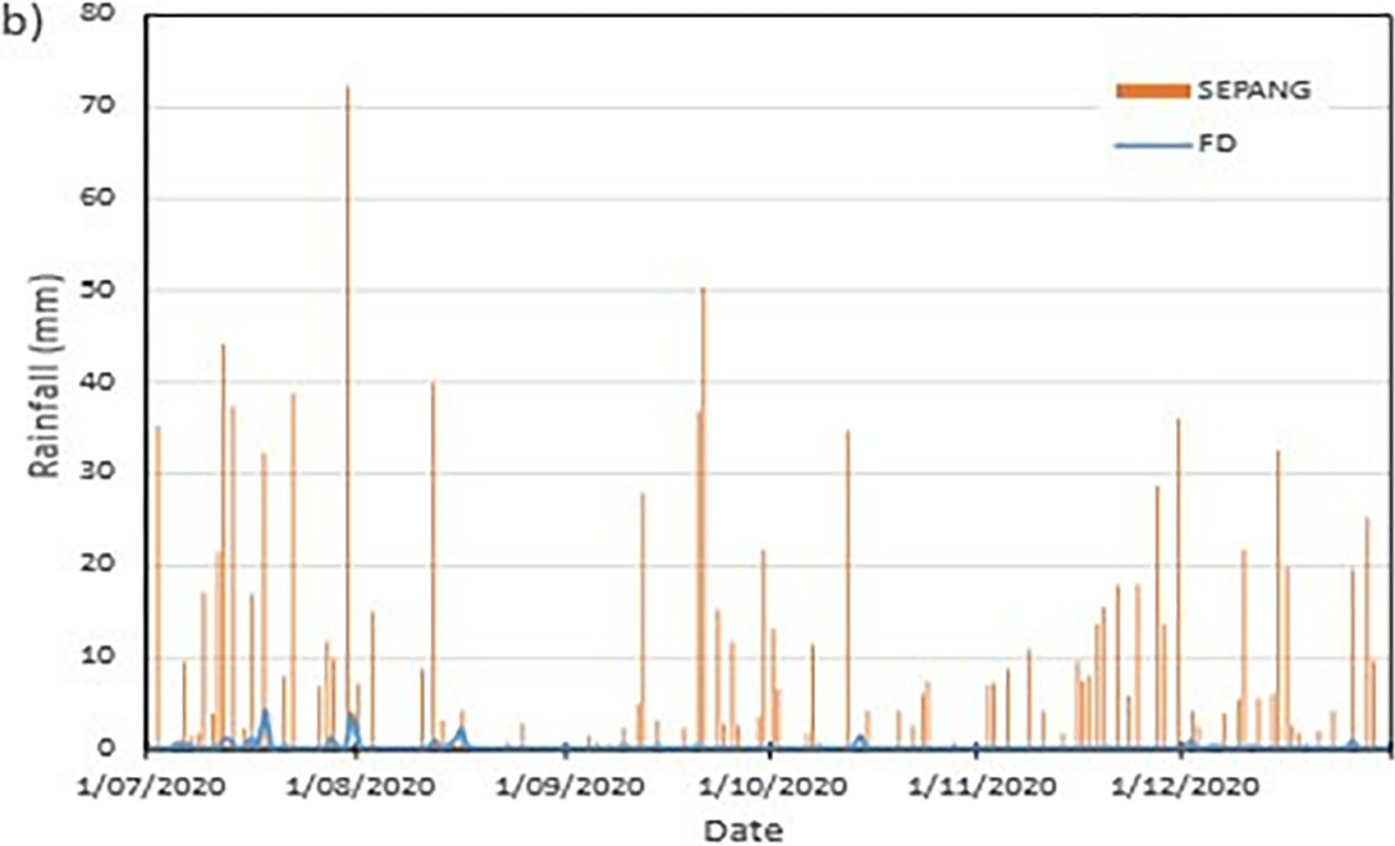
Precipitation records of KLIA Sepang in comparison to the field monitoring.

Another set of data from the nearest weather station during the period of the field monitoring was collected and illustrated in **Fig 7.** The data was obtained through Malaysian Meteorological Department as an additional data to compensate the impaired data from the field monitoring. The data obtained shows the rainfall event is comparable to the records obtained from the field monitoring. The rainfall event obtained from the site occurring during the months July and August 2020 coincides befittingly with the rainfall event occurring at the Sepang KLIA weather station.

From the data obtained the highest amount rainfall total was during July with 371.4 mm in total with 20 rainfall events. The month following it is November with 214 mm in total of the 19 rainfall events occurring. The driest month according to the data is August with 82.2 mm of rainfall in 12 rainfall events. The most intense rainfall event during the recorded period is on 30 the of July with a precipitation amount of 72.2 mm in a day.

From the data obtained the highest amount rainfall total was during July with 371.4 mm in total with 20 rainfall events. The month following it is November with 214 mm in total of the 19 rainfall events occurring. The driest month according to the data is August with 82.2 mm of rainfall in 12 rainfall events. The most intense rainfall event during the recorded period is on 30 the of July with a precipitation amount of 72.2 mm in a day.

**Fig 8:**
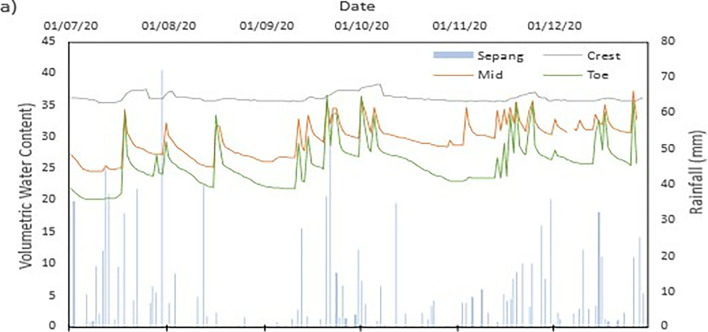
The volumetric water content for depth 1 m.

**Fig 9:**
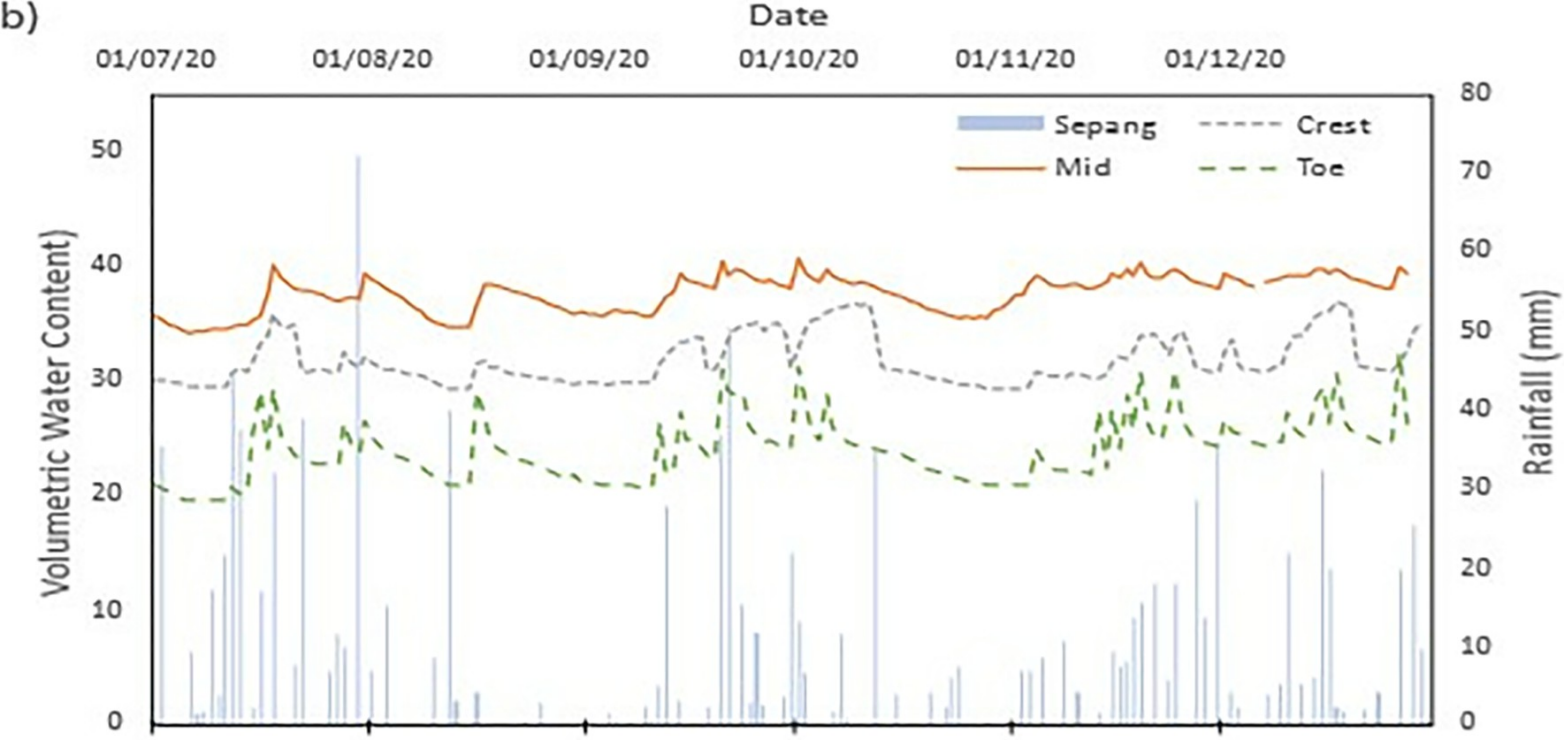
The volumetric water content for depth 0.5 m.

**Fig 10:**
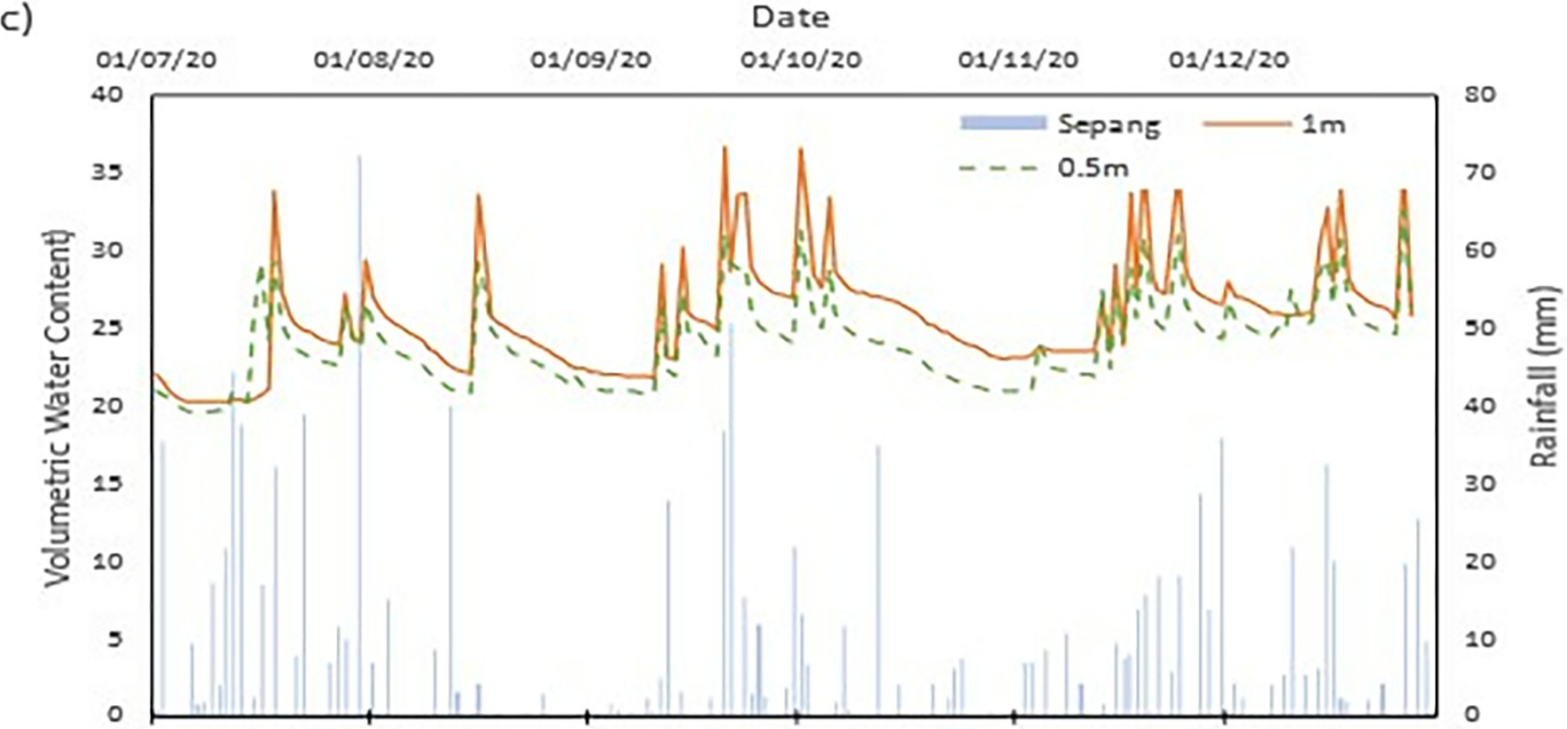
The volumetric water content for toe section.

**Fig 11:**
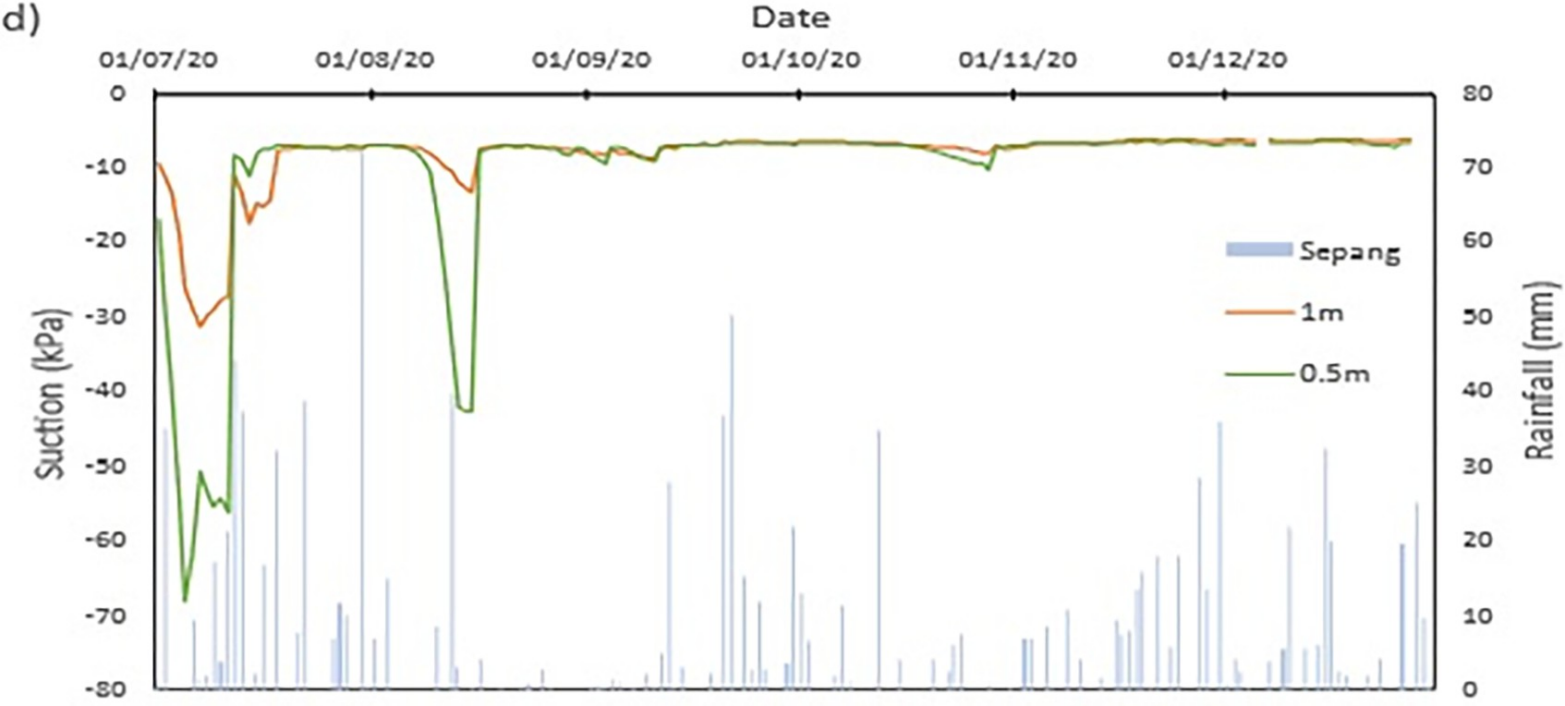
The volumetric water content for middle section.

**Fig 12:**
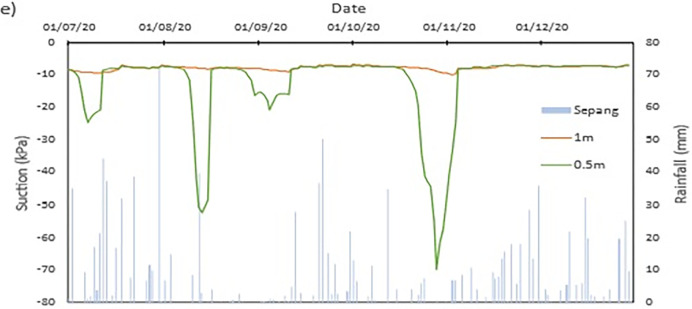
The volumetric water content for crest section.

**Fig 13:**
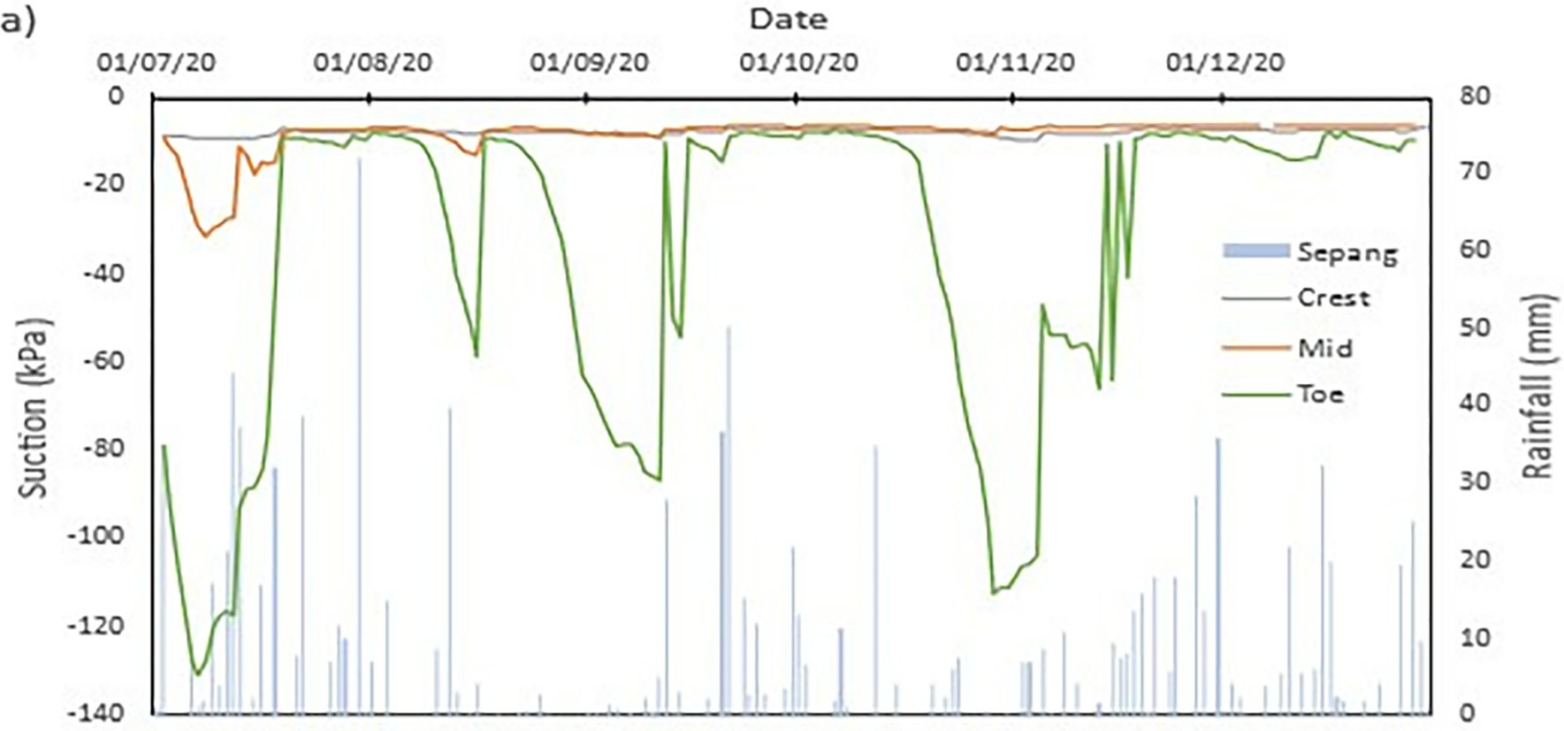
The matric suction for depth 1 m.

**Fig 14:**
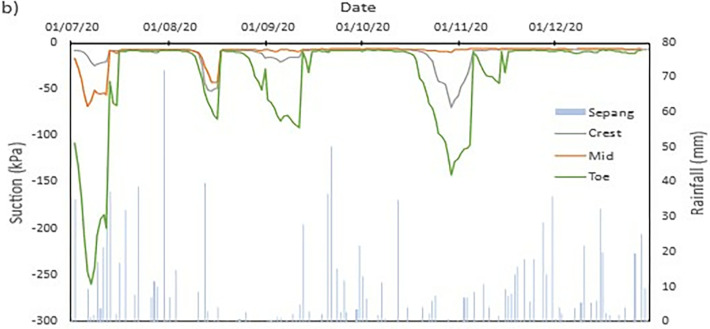
The matric suction for depth 0.5 m.

**Fig 15:**
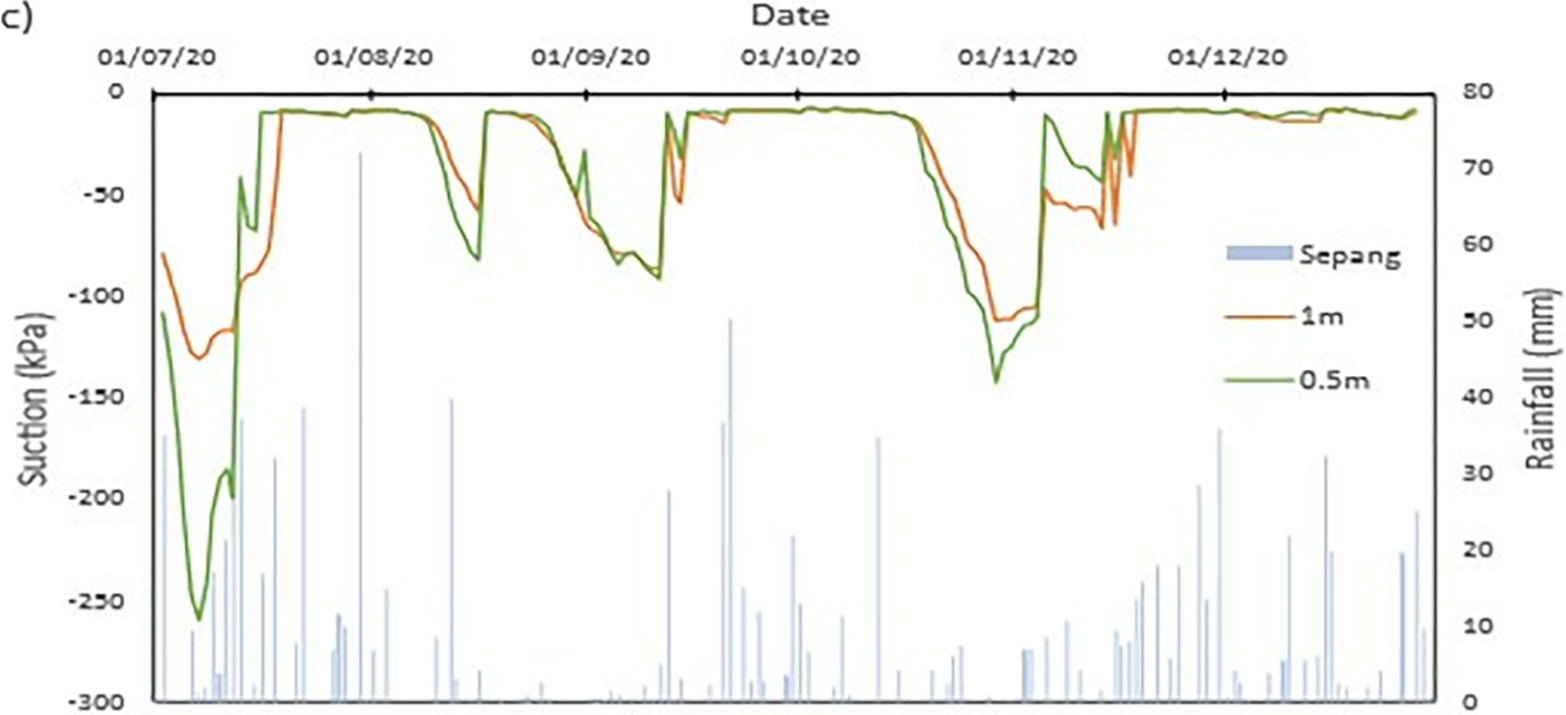
The matric suction for toe section.

**Fig 16:**
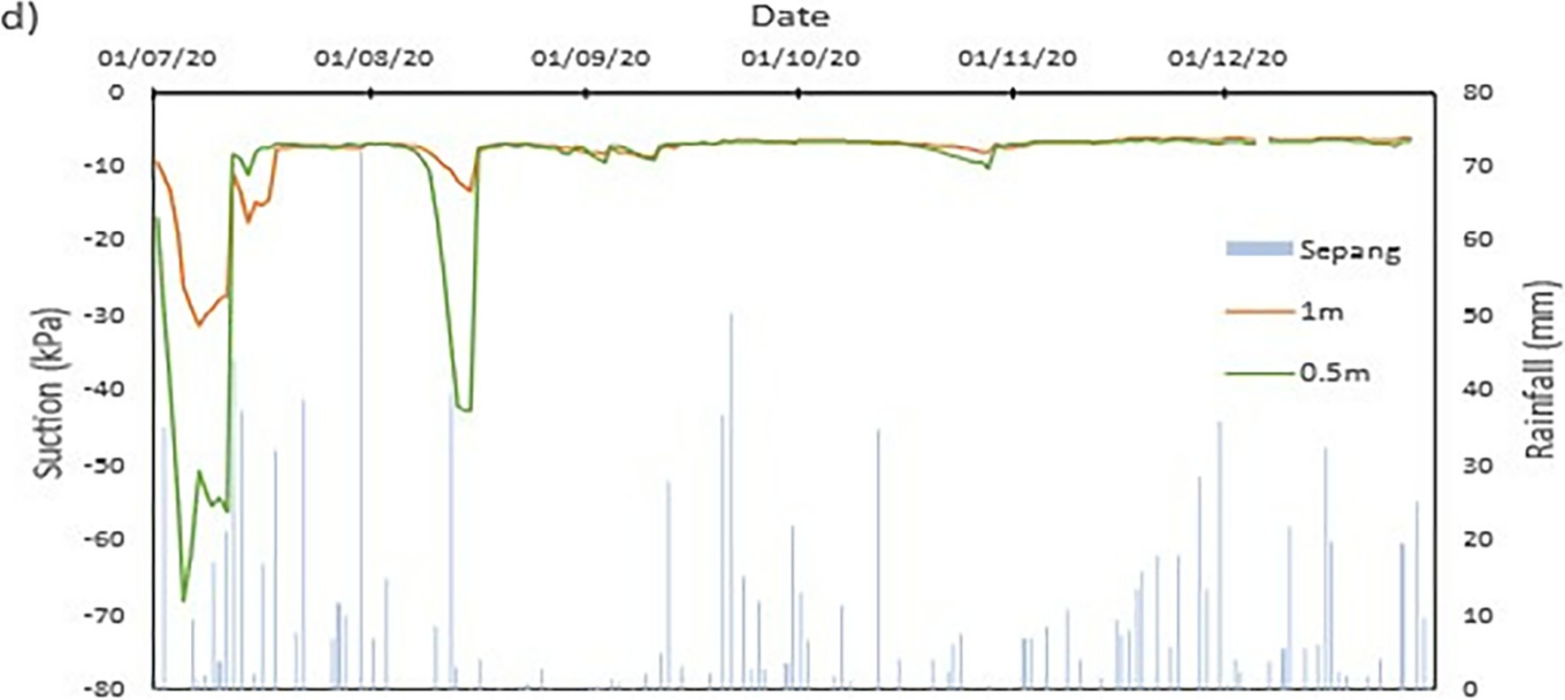
The matric suction for middle section.

**Fig 17:**
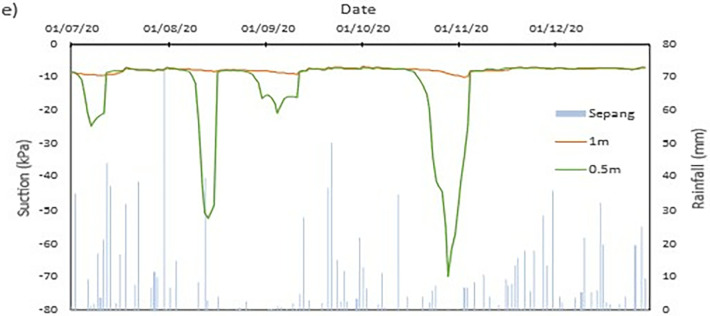
The matric suction for crest section.

The data set obtained from KILA Sepang is comparable to the recorded data of the field monitoring. This can be seen as most of the peak value from the field monitoring coincide nicely with the second data set. Even though the value of the data differs; the data has a high degree of agreeableness based on the trend of the timing of rainfall and highest amount moment, which also align well with regional precipitation patterns. It can also be observed in **Figs 8–17**, the rainfall event from the Sepang KLIA records does coincide suitably with the reaction obtained from the sensors for both the volumetric water content and suction on the slope.

#### b) VWC vs time graph

The VWC values at the crest site for a depth of 1 meter, as shown in Fig 8, range from roughly 0.36 m^3^/m^3^ to 0.39 m^3^/m^3^, suggesting a comparatively steady moisture content that is less affected by surface infiltration than at lower slope positions. The mid-slope location’s VWC, on the other hand, changes more dramatically, ranging from 0.24 m^3^/m^3^ to 0.37 m^3^/m^3^. The big range in VWC suggest that the area is more susceptible to rainstorm events and that there may be horizontal drainage along the slope profile. The VWC values at the toe of the slope range from 0.20 m^3^/m^3^ to 0.36 m^3^/m^3^, which represents the buildup of surface runoff and drainage patterns common in lower slope areas.

As illustrated in Fig 9 for shallower depth of 0.5 meters, the VWC at the crest position ranges from 0.29 m^3^/m^3^ to 0.37 m^3^/m^3^, but the mid-slope location sees a higher range from 0.34 m^3^/m^3^ to 0.41 m^3^/m^3^. While both have comparable range, it can be deduced that the crest has more numerous small inter particle pores than mid-slope section which allow higher water content. At the toe location, the VWC values range from 0.19 m^3^/m^3^ to 0.33 m^3^/m^3^, Similar range observed from the deeper depth of 1 meter which but slightly lower. The lowest VWC measured was at the toe location for both the 0.5 m and 1 m depths which is 0.19 m^3^/m^3^ and 0.20 m^3^/m^3^, respectively. This indicates that the soil at these locations has a comparatively poorer capacity to retain moisture during dry spells. However, the mid-slope and crest locations record the highest VWC values of 0.41 m^3^/m^3^ and 0.39 m^3^/m^3^, respectively, indicating regions where water tends to accumulate or where soil has better moisture retention.

The range obtained from both depths does not stray too far from each other. This indicates that the water content of the soil of the region is less moist and does not reach saturation easily during a singular rainfall event. From **Figs 10–12** it can be seen that the 0.5 m depth sensors respond rapidly to rainfall intrusion in comparison to the 1 m depth sensors lagging in responsiveness. As the water infiltrated into the soil will need longer time to reach the deeper sensor, thus producing the results produced. The surrounding area is covered with vegetative growth that acts as a shelter for the rainfall to reach the soil while also being a negative water pressure due to the root of the vegetation absorbing the water in the soil [38]. It is an indication of the gradual reduction of the impact of external conditions on deeper soils [38, 42, 43].

Zhang et al. [62] conducted a study in field monitoring in a field in southern Jiangsu, China, that is also in subtropical region, which installed suction and water content measurement at very shallow depths of 0–10, 10–20, 20–40, 40–60 and 60–80 cm. At each depth, the water content ranged from 0.184 to 0.458 m^3^/m^3^, 0.23 to 0.400 m^3^/m^3^, and 0.236 to 0.433 m^3^/m^3^, 0.393 to 0.447 m^3^/m^3^ and 0.369 to 0.486 m^3^/m^3^ in that order. They concluded that the higher water content at deeper depths was caused by strong precipitation events and the constant replenishment of moisture from shallow groundwater, whereas the shallower depth range of 0–40 cm demonstrated a quick reaction to changes in water content brought on by evaporation and rainfall. The water content range that the field monitoring system obtained is in good agreement with the silty clay loam research conducted by Zhang et al. [62].

#### c) Suction vs time graph

The negative pore water pressure, also known as matric suction, is represented in Figs 13–17. At depth of 0.5 m, the range of matric suction obtained for the Toe is between 255 kPa to 7 kPa, while at the mid-slope is between 70 kPa to 7 kPa, and at the crest the range of the matric suction acquired is between 70 kPa to 7 kPa. For the depth of 1 m, the matric suction recorded for the toe is at a range of 130 kPa to 7 kPa, at mid-slope is at the range of 30 kPa to 7 kPa, and at crest is at a range of 10 kPa to 7 kPa. From the data obtained, it can be surmised that the limit of the lower end of the matric suction recorded is the range of the air-entry value for the soils on site.

The pore pressure at the toe region is more fluctuating in bigger range in comparison to the rest of the location for both depths. This is due to the toe is closer to a big, rooted tree even though there is less vegetation at the toe of the slope. From the **Figs 10–12**, the sensors at depth 0.5 m have a more immediate reaction and fluctuating to the surrounding environment while the 1m depth is more progressive in comparison which has been also corroborated from experiment done by Crawford et al. [63] and Haris et al. [41]. This observation can be seen true for all the locations on the slope. One of the reasons is due to the vicinity of sensors at depth 0.5 m to the roots of trees and vegetation. The roots of the tree function as a vacuum, creating negative pore pressure while absorbing the moisture in the soil around it [38]. The region has also high sunlight exposure thus accelerating the evaporation occurring on the soil and the water droplet on the plants from reaching the ground, hence being the sensors that is closer to the surface of the soil will have the surrounding moisture to be more readily evapotranspiration through the capillary fringe of the soil. This in turn creates an environment of high negative pore water pressure near the surface of the soil.

Zhang et al. [62] also recorded the suction at different depths from 0 cm to 80 cm, which has a range of 10 kPa to 1200 kPa during a wetting cycle while 10 kPa to close to 10000 kPa during a drying cycle. The suction measurement recorded was less reactive in comparison to the VWC measurement which is similar to data collected from the study done in this paper. The change in soil suction Zhang et al. [62] recorded was relatively stable in comparison to this paper recorded data but was consistent with the variation in suction and soil water content.

#### d) VWC vs matric suction

**Fig 18:**
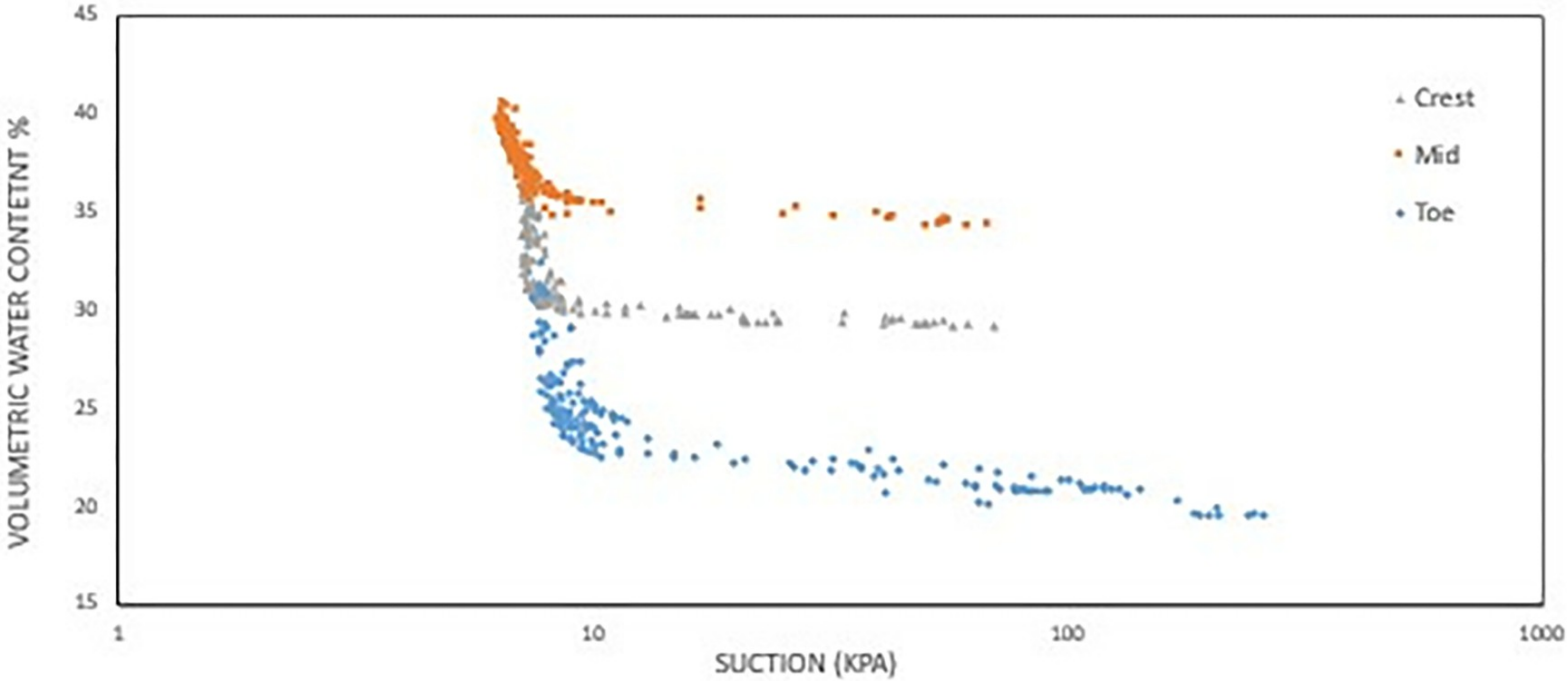
Data chart for volumetric water content vs matric suction at 1 m depth.

**Fig 19:**
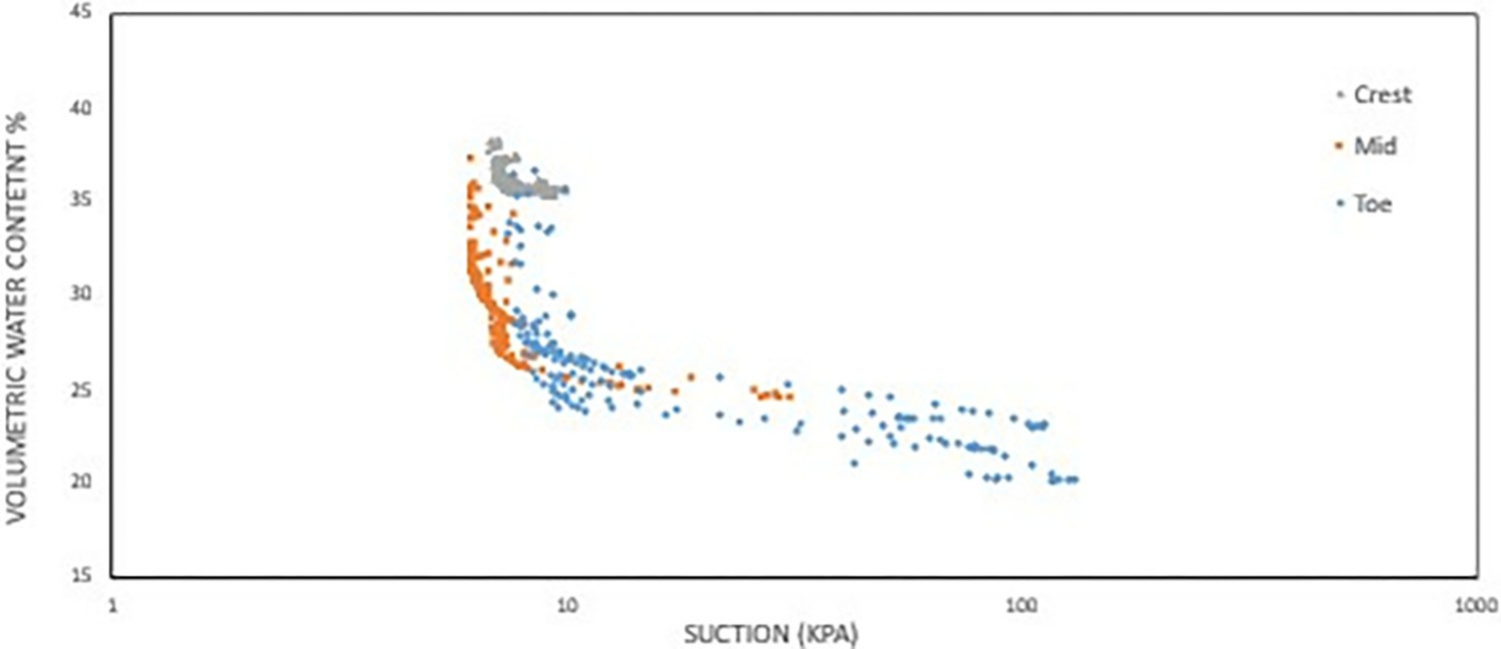
Data chart for volumetric water content vs matric suction at 0.5 m depth.

Data chart of the volumetric water content against the water potential (matric suction) was formed from the data collected as from both sensors combined in Figs 18 and 19 which provides insights into how water is retained in soil pores under varying conditions. In soil structure interface, the effective saturation (VWC) usually falls as the suction stress increases and vice-versa, which can be seen observed in the chart produced in Figs 18 and 19. We can observe that the trendline of the recorded data for both Figs 18 and 19 formed a partial sigmoidal pattern which is synonymous to a SWCC.

For **Fig 18** which is at depth of 1 m, the range of water content for both the mid and crest section is quite similar to each other, but the crest having a much smaller range of water content while also having a higher saturated and residual water content value. The data obtained for the 0.5 m depth shows the range and trend for all the data is similar. The only differences between them are the residual and saturated water content, as the highest values for both is the mid-section, following the crest which has intermediate values, and the lowest values is the toe section. Because the soil structure varies throughout the slope, this stratification highlights the disparities in moisture retention abilities between location even more.

The figure shows that for both 0.5 m and 1 m depths all have similar air-entry value based on visual observations on the graph which is around the range of 7 kPa to 9 kPa. For 1 m depth the toe section has the higher AEV followed by crest and lastly the middle section. For 0.5 m depth, all three have similar AEV with slight difference of margin. Meanwhile, the toe section has the highest AEV followed by the crest and then the mid-section which is similar ranking to 1 m depth. Higher AEV signifies better ability of water retention in the soil and finer macro-pores making the toe section having the best water retention with the crest and middle section followed respectively. **Figs 18 and 19** data charts will be used as a baseline comparison for obtaining the fitting parameters for the related predictive curve models.

### Performance evaluation for best fit equation

As the SWCC is a nonlinear function, the values of each parameter were fitted using the least squares method. The fitting degree was measured using two methods which are the Root Mean Square Error (RMSE) and regression models evaluation metric, (R^2^). The Root Mean Squared Error (RMSE) is a performance indicator for a regression model. It is the square root of the average of the set of squared variations between coordinated values from identical sites in the dataset along with coordinate values from an alternative source. It provides an estimation of how accurately the model can estimate target value or positional precision. The equation used for the RMSE is shown in Eq 4:

$$RMSE=\sqrt{\sum_{i=1}^{n}\frac{{\left({\hat{y}}_{i}-y_{i}\right)}^{2}}{n}}$$ (4)

The RMSE is a popular evaluation tool for regression issues due to it not only determines the average accuracy of the prediction but also highlights the impact of significant errors which value will be impacted by large mistakes. The model and its forecasts are more accurate for this performance indicator the lower the matric number. A higher RMSE suggests a significant difference between the residual and the source dataset. If the test sets RMSE is significantly higher than the source sets, it means the fitting parameters have severely overfit the data. This shows the model created will have low predictive value when tested out of sample.

Another performance measurement is the regression models evaluation metric, R^2^ which is the coefficient of determination for proportion of variance in the linear regression relationship between the observed data variables and model anticipated values. The model is valued at how close the value of R^2^ to one, as the closer to it is better. A common misperception about regression modelling is that a low R-squared score is always an undesirable outcome. The equation of the regression model is shown below:

$$R^{2}=1-\frac{\sum_{i=1}^{\Pi }{\left(y_{i}-{\hat{y}}_{i}\right)}^{2}}{\sum_{i=1}^{\Pi }{\left(y_{i}-{\bar{y}}_{i}\right)}^{2}}$$ (5)

### Result of model fitting

**Fig 20:**
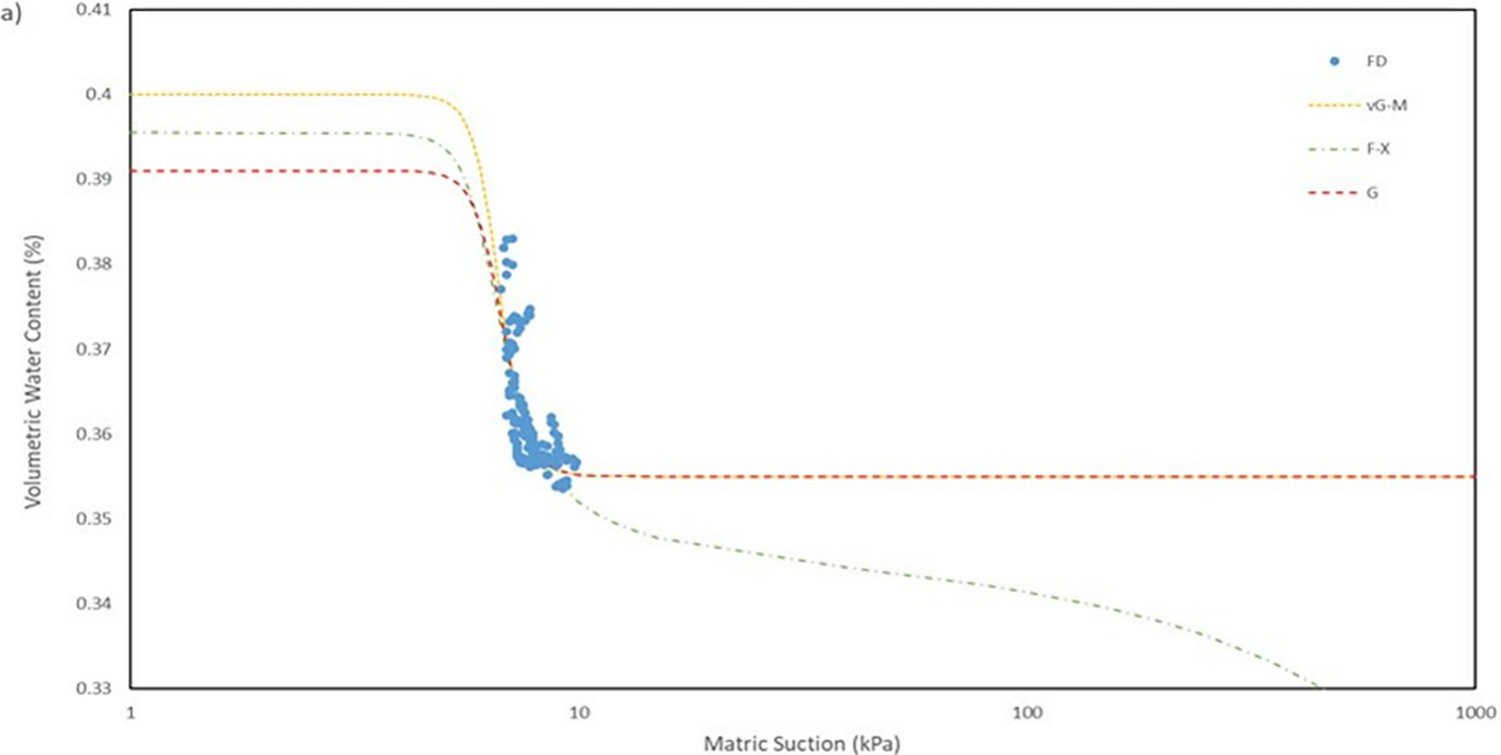
SWCC fitted using field monitoring data for location points at 1 m depth crest section.

**Fig 21:**
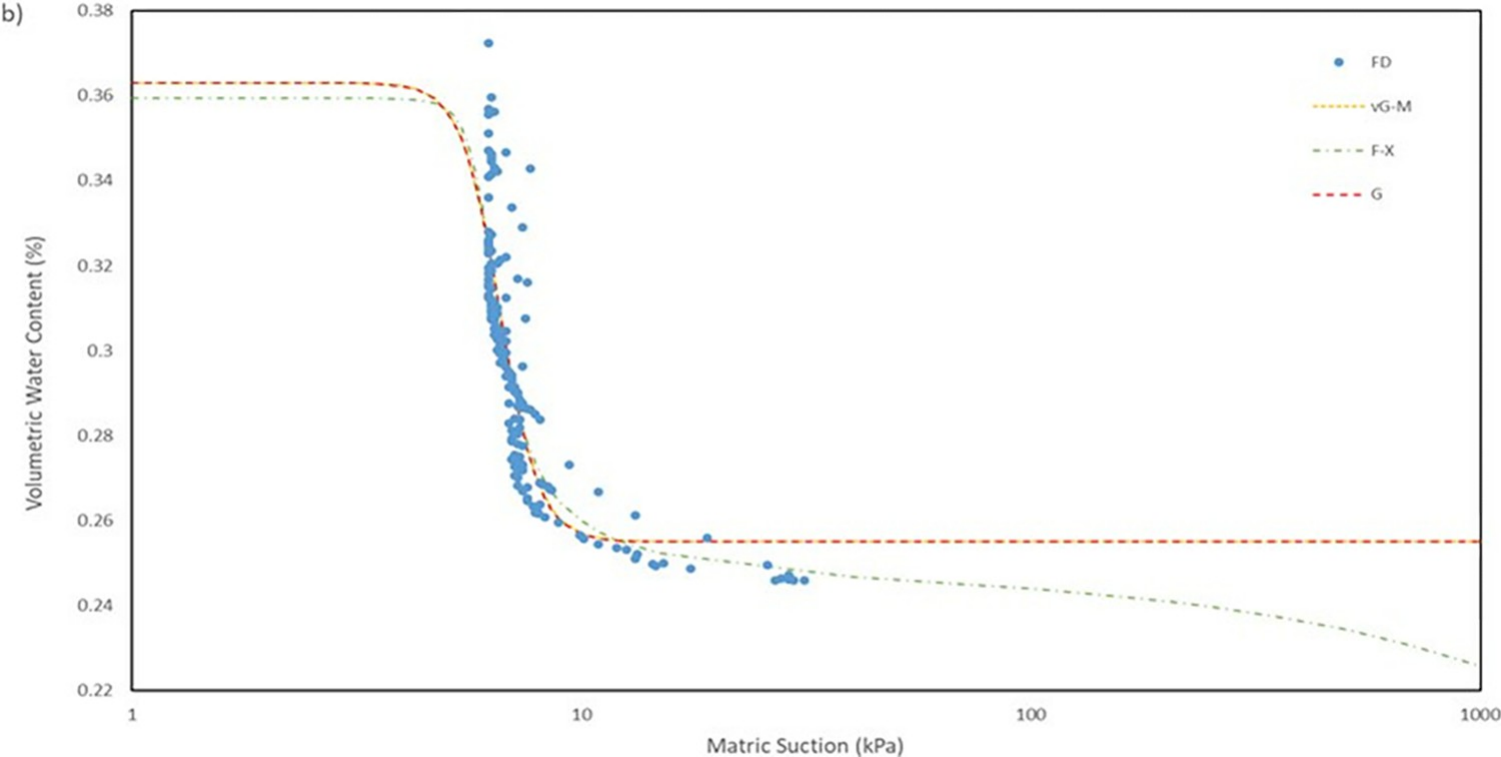
SWCC fitted using field monitoring data for location points at 1 m depth middle section.

**Fig 22:**
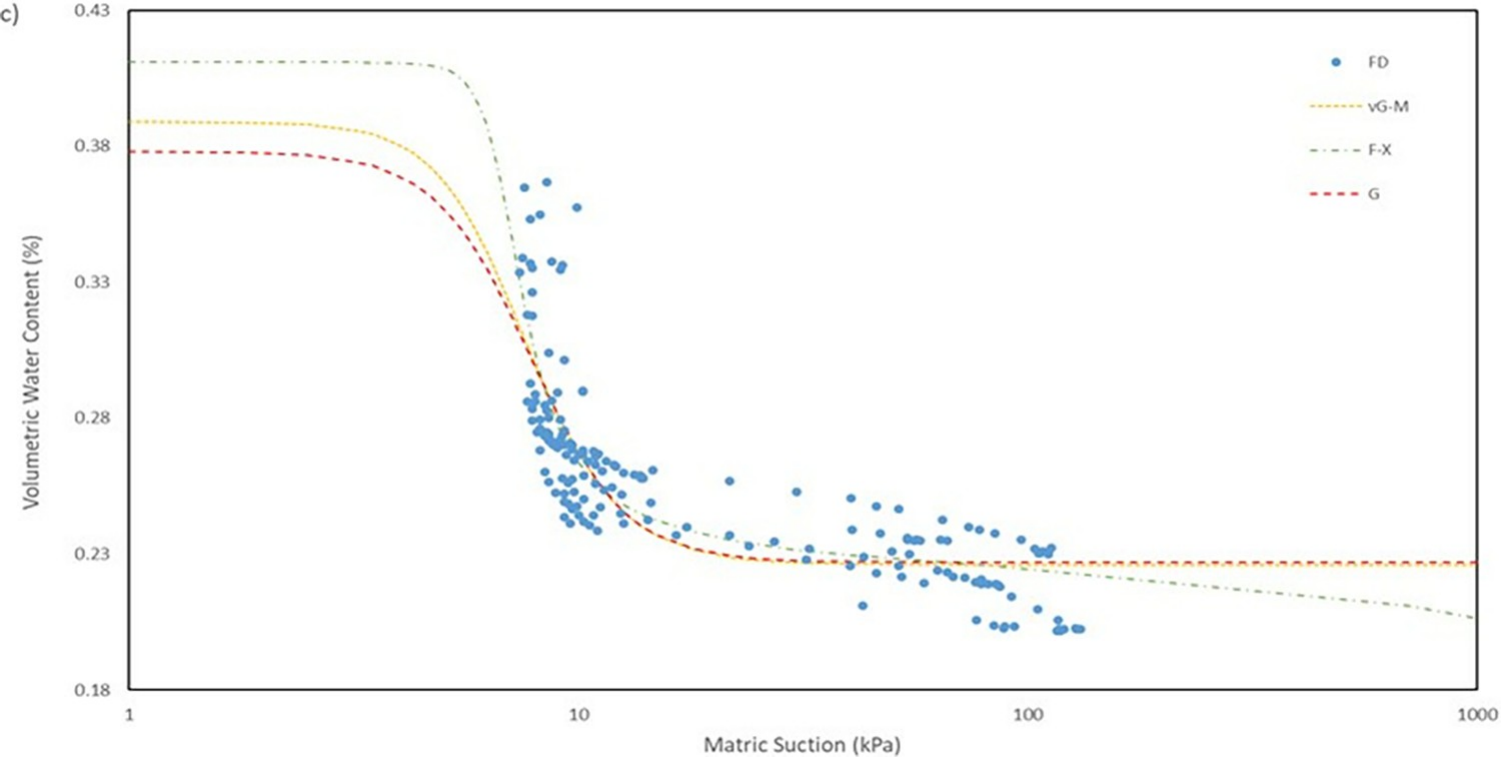
SWCC fitted using field monitoring data for location points at 1 m depth toe section.

**Fig 23:**
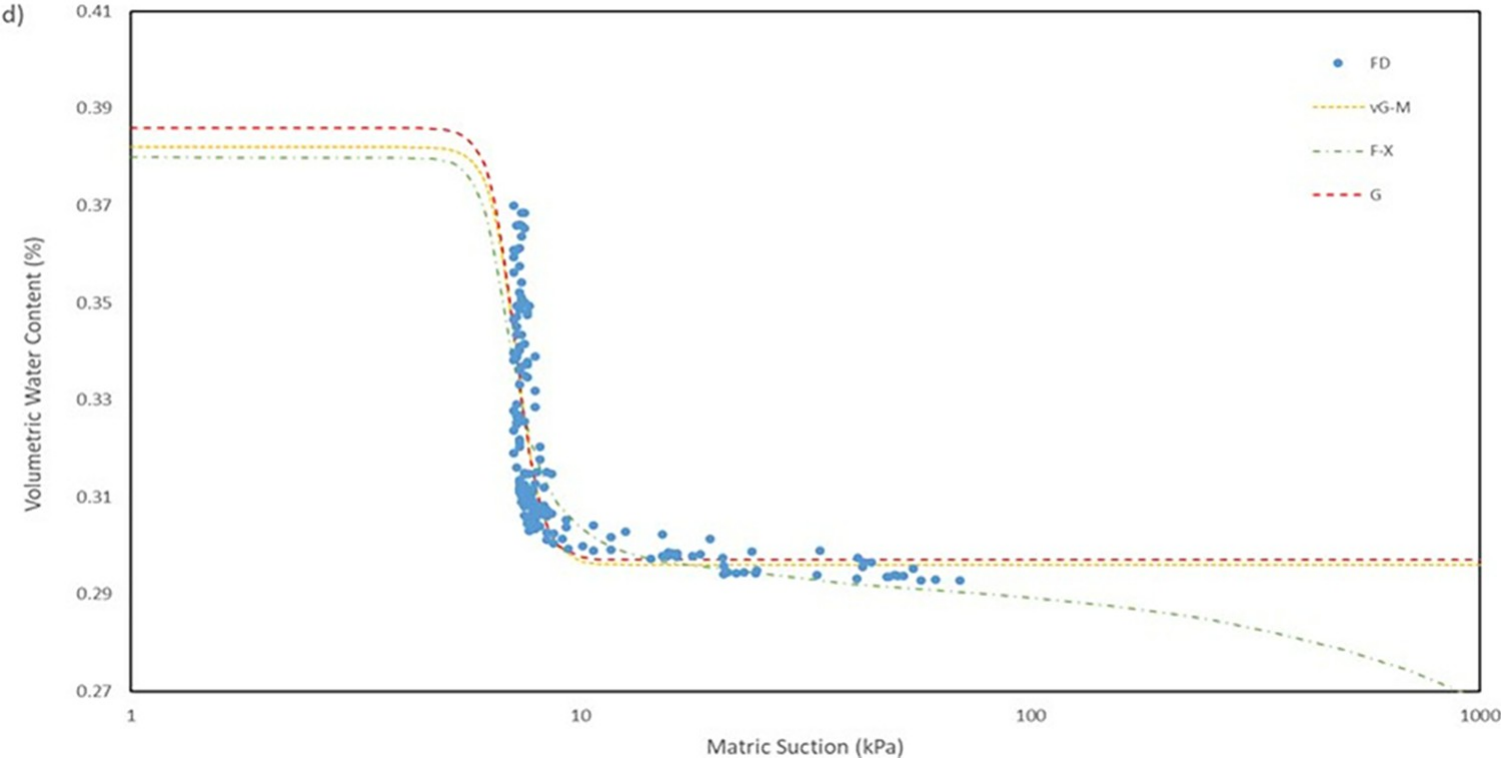
SWCC fitted using field monitoring data for location points at 0.5 m depth crest section.

**Fig 24:**
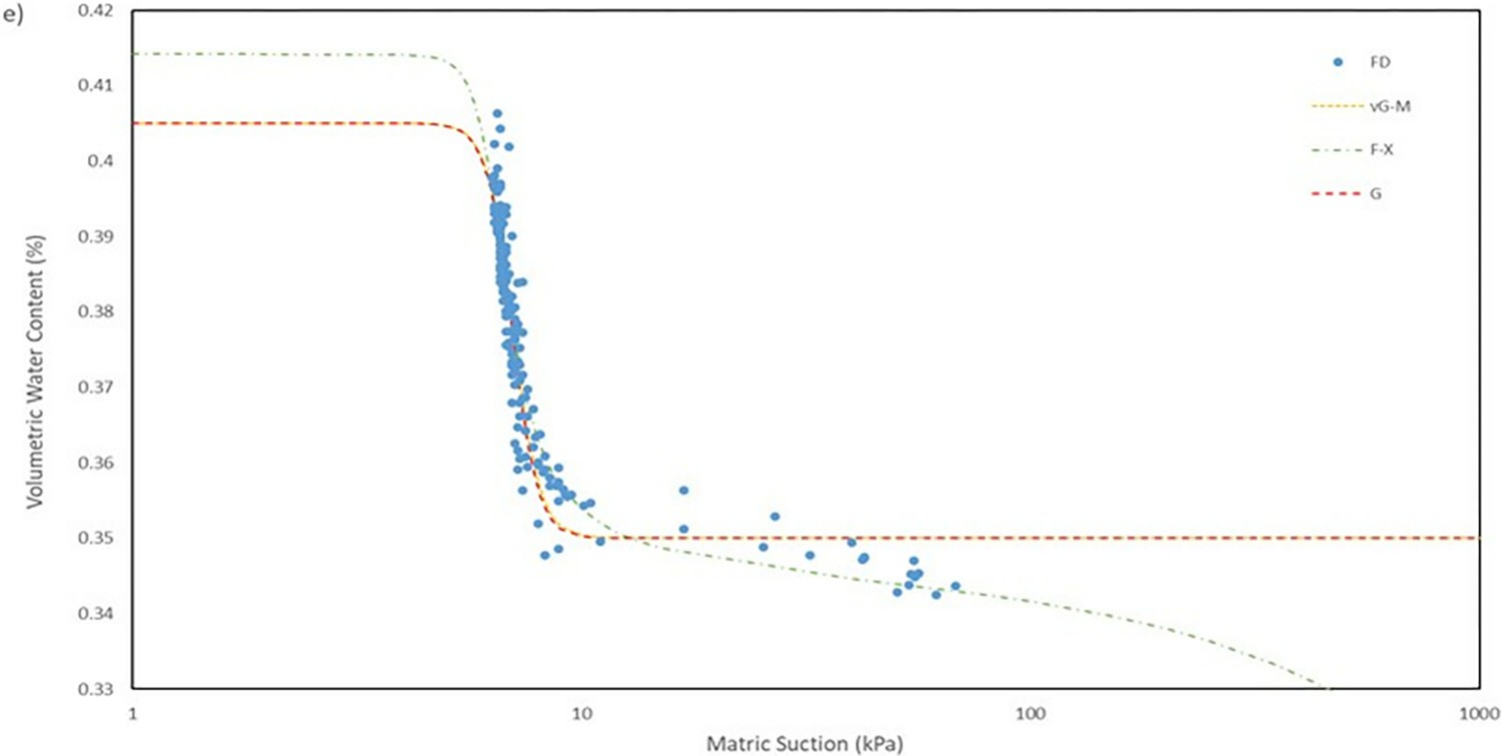
SWCC fitted using field monitoring data for location points at 0.5 m depth middle section.

**Fig 25:**
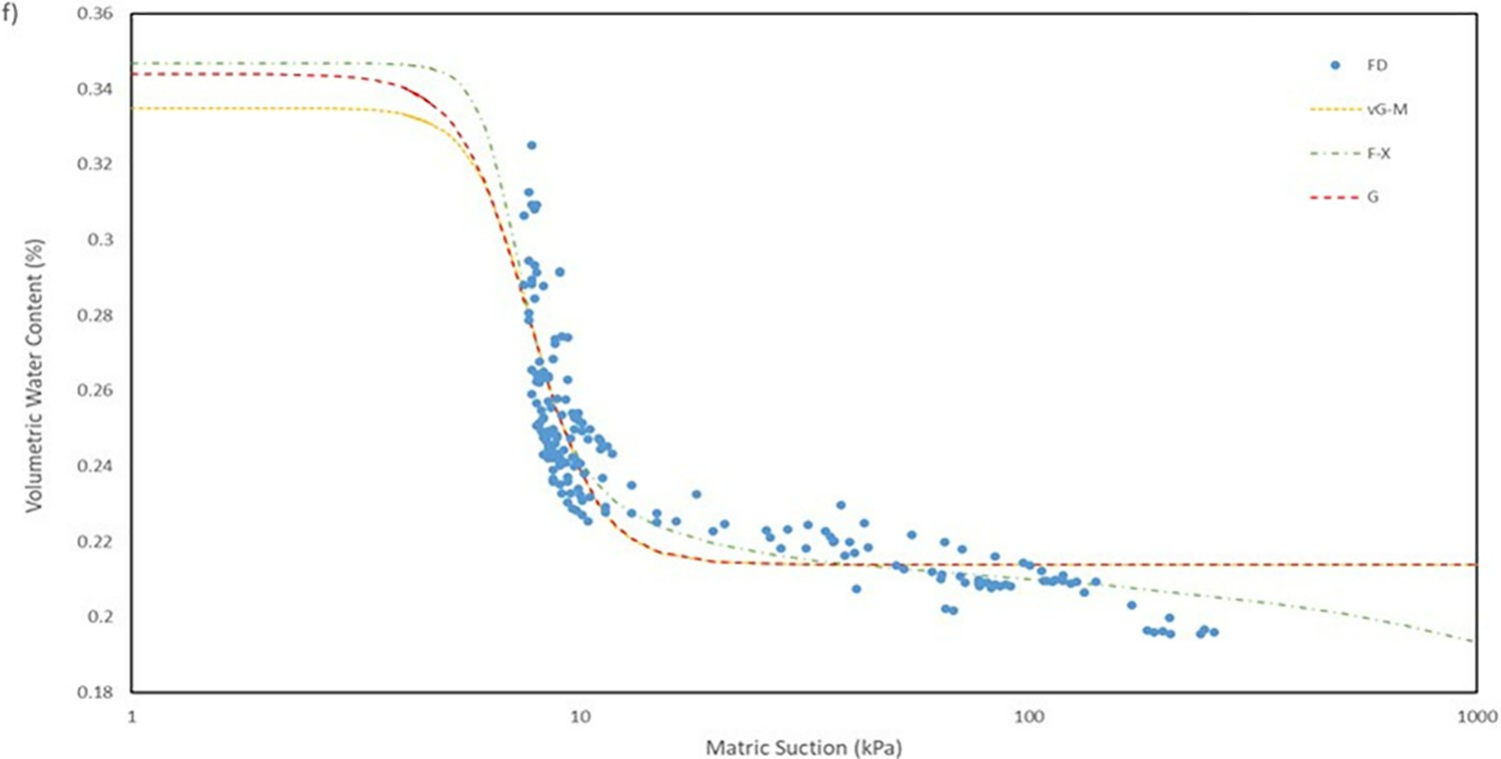
SWCC fitted using field monitoring data for location points at 0.5 m depth toe section.

For the model used for the fitting parameters, three were chosen which are van Genuchten-Mualem variation (vG-M), Fredlund-Xing model (F-X) and Gardner (G) as shown in **Table** 4. These three statistical models are governed by 4 to 5 independent parameters which are used to fit the model for the soils. All the data was fitted within a constraint as to mitigate incompatible fitting, which will restrict their variation to a predefined range. The observed data and the fitted model curves are contrasted in **Figs 20–25** to further highlight the behavior of the three models investigated in this paper when fitted to the SWCC data for different soil samples.

**Table 4:** Fitting parameters values and RMSE for different depth and location of soil.

Position	Depth (m)	Model	a	n	m	θ_r_	θ_s_	R^2^	RMSE	Weightage
Crest	0.5	vG-M	7.15	15.0	0.933	0.296	0.382	0.5699	0.01377	2
		F-X	6.49	15.0	1.000	0.290	0.380	0.5315	0.01437	1
		**G**	**7.15**	**10.0**	**-**	**0.297**	**0.386**	**0.5703**	**0.01376**	3
	1.0	vG-M	6.59	14.8	0.932	0.355	0.400	0.4557	0.00468	3
		F-X	6.07	12.4	0.950	0.343	0.388	0.4168	0.00484	1
		**G**	**6.75**	**13.6**	**-**	**0.355**	**0.391**	**0.4578**	**0.00467**	2
Mid	0.5	**vG-M**	**7.01**	**15.0**	**0.933**	**0.350**	**0.405**	**0.8993**	**0.00504**	2
		F-X	6.27	15.0	0.950	0.343	0.405	0.8968	0.00510	3
		G	7.01	15.0	-	0.350	0.405	0.8988	0.00505	1
	1.0	vG-M	6.50	10.2	0.902	0.255	0.363	0.9540	0.01454	1
		**F-X**	**6.05**	**15.0**	**0.950**	**0.243**	**0.347**	**0.9951**	**0.01436**	3
		G	6.60	9.9	-	0.255	0.363	0.9523	0.01460	2
Toe	0.5	vG-M	7.63	6.4	0.844	0.214	0.335	0.7496	0.01363	2
		**F-X**	**6.71**	**10.0**	**1.000**	**0.207**	**0.347**	**0.7832**	**0.01268**	3
		G	7.70	5.5	-	0.214	0.344	0.7474	0.01369	1
	1.0	vG-M	7.09	4.6	0.783	0.226	0.389	0.6589	0.02040	2
		**F-X**	**6.71**	**11.8**	**0.950**	**0.220**	**0.395**	**0.6924**	**0.01935**	3
		G	7.82	4.1	-	0.227	0.378	0.6553	0.02050	1

The fitted data performance was evaluated with the use of the RMSE, a performance evaluation tool for a regression model, R^2^ which is the coefficient of determination for proportion of variance. The statistical values were further assisted with the weightage given for each model fitting, to determine which model gave the overall best performance in modelling the SWCC of the given soils. In the table, the weightage of three are awarded to the model with the smallest RMSE and highest R^2^ value which represents the best fit in the group. A smaller weightage is given to the followed best and the worst performed model is given a weightage of one as it has the biggest value of RMSE. The greater the cumulative weightage achieved, the more effectively the model aligns with the collected field monitoring data. The data used is provided in S1 File.

Utilizing the weightage presented in **Table 4**, it is evident that the vG-M model outperforms the other models, accumulating a total weight of 13 out of a total of 18. The next best fitted model is F-X model with a weight of 12 and followed by the G model with a weight of 11, which is the lowest weight obtained. The model fitted well in most of the soils data collected. Based on the performance indicator, RMSE, it is evidently indicated that the model vG-M has the best fit for most soils samples except for soil samples located at the crest for both depth of 0.5 m and 1.0 m as even when it is not the best fitted it still lag by a tiny margin in comparison to the lead. The range of RMSE value for the model vG-M is between 0.02040 to 0.00468, while model F-X and G are 0.01935 to 0.00484 and 0.02050 to 0.00467, respectively. Even though model G has the lowest weightage, its performance is not lagging behind the other models as the difference between the values of RMSE is minor.

The RMSE is a popular evaluation tool for regression issues due to it not only determines the average accuracy of the prediction but also highlights the impact of significant errors which value will be impacted by large mistakes. The model and its forecasts are more accurate for this performance indicator the lower the matric number. A higher RMSE suggests a significant difference between the residual and the source dataset. If the test sets RMSE is significantly higher than the source sets, it means the fitting parameters have severely overfit the data.

The R^2^ for the entire model fitted is in the range of 0.41 to 0.99 which considered to be a broad range. The R^2^ value for each position has its own range, as can be observed seen for position at crest for 1.0 m depth has a value range of 0.41 to 0.46, which is the lowest range from the data compiled, and position mid at 1.0 m depth with a value range of 0.95 to 0.99, indicating that the model was fitted exceptionally well. The low number range at the crest for both 0.5 m and 1.0 m depth even though by eye observation, the model line fitted nicely on the data in **Fig**s 20–23 is due to the collection of mass data obtained from the field measurement which is concentrated in variant part of the graph, as a single spot can be taken place by numerous of the same data. This was demonstrated by comparing the R^2^ obtained from crest 1.0m with the RMSE calculated. The R^2^ calculated with the mean of 0.44 is the lowest among the overall data fitted but it also has the best RMSE value with mean of 0.00473 whereas the location mid at depth of 1.0 m have the highest R^2^ with mean of 0.97 has an average value of RMSE of 0.0145 which is the middle of the group that was fitted.

This indicates that R^2^ measurement for model performance is highly reliance on the dispersion of the variables or data set collected. As the R^2^ is measuring on the precision of the data collected as opposed to the accuracy which R^2^ has been criticized as a reliable indicator of model performance when forecasting a binary outcome, both because its value is frequently low and because it is sensitive to the distribution of the event under consideration. R^2^ as a measurement model is excessively sensitive to extreme values and cumulative variations between model predictions and recorded data [64, 65]. The RMSE produced by the fitting models are observed higher than usual, this is due to the data set collected from the field monitoring system is used in comparison to lab test equipment such as pressure plate test.

In a linear data collection, for instance in a regulated or controlled environment, especially a laboratory, the data obtained can be more precise and linear, this will assure a more well fitted model if using the R^2^ regression model as an evaluation metric. The pressure plate test would produce a linear outcome of data set for each point of data in comparison to the data collected by a field monitoring system which are affected by multitude of factors such as sunlight concentration, rainfall intensity, vegetation abundancy and wind strength.

It can be observed that the fitting errors associated with the vG-M model are comparatively lesser than those of the alternative models. This discrepancy is attributable to the superior depiction of vG-M model for the given soil samples of the slope. For F-X model, it can be seen to be more fitting for a continuous decline of the water content at higher matric suction after reaching the residual zone. This can be exemplified in the SWCC generated in **Fig**s **20–25**, as the water content keeps declining at high suction matric even below the residual water content set in the fitting model. As most of the data collected shows that the water content continues to drop after the transition zone. In comparison to vG-M and G models, the residual water content is relatively constant with very minimal reduction. This is due to the mathematical equation of the model being simpler with lesser fitting parameters is the limiting factor for the model to allow an accurate depiction of the soil’s water content at very high matric suction.

Due to the sensors’ inability to detect effectively at exceedingly high matric suction and at zero matric suction, the data obtained for the SWCC is limited. Due to these restrictions, the fitting model can only be utilized with data that was mostly collected during the transitional zone and the lower matric suction of the residual zone. The sensors are able to detect the water content during the air-entry value, but it would be unreasonable to prove this with absolute certainty. Naturally, fitting SWCC with a wider or complete suction range would considerably reduce the variances among the models, but obtaining ideal conditions and the optimal equipment for field monitoring would be impractical. Even though the data acquired through the field monitoring is limited in the range of matric suction that can be observed, it is sufficient data to perceive the SWCC trendline forming from it and a proper fitting curve can be formed. It is also the most direct way of finding the SWCC through the natural phenomena itself occurring on site producing a more complex system of factors affecting the matric suction in soil [66]. Researched was conducted on the models created based on Fredlund and Xing’s (1994) best fit SWCC equation were determined to be the least sensitive to the SWCC suction range following a sensitivity study by Rahimi et al. [67]. Thus, it is recommended to employ Fredlund and Xing (1994) based models for the calculation of the SWCC data if a wide suction range is not available while the Van Genuchten model is commonly utilized because to its excellent fitting accuracy and obvious physical interpretation of the parameters. [68].

## Conclusion

In conclusion, the six sets of SWCC obtained from six samples of soil at different parts and depth of the slope were collected and studied in this literature. The statistical measure, RMSE and the regression model, R^2^ was used to evaluate the curve fitting for the soil database obtained from the field monitoring between the three models chosen which are van Genuchten-Mualem variation, Fredlund-Xing and Gardner. The results show that for crest section of depth 0.5 m and 1.0 m the best fit is Gardner for both with RMSE of 0.01376 and 0.0467, and R^2^ value of 0.5703 and 0.4578 respectively. Meanwhile for the middle section the best fit for depth of 0.5 m is van Genuchten-Mualem model with RMSE of 0.00504 and R^2^ value of 0.8993 while at 1.0 m depth is Fredlund-Xing model with RMSE of 0.01436 and R^2^ value of 0.9951. For the toe section of the slope, the best fit model for 0.5 m and 1.0 m are both Fredlund-Xing’s with RMSE of 0.01268 and 0.01935, and R^2^ value of 0.7832 and 0.6924 respectively.

The inference regarding which model offers the best or worst estimation, based only on RMSE values, is that it depends on the soil database and varies for different databases and from the study done it was observed that Fredlund and Xing’s (1994) provide the best fitting for the majority of the samples, however van Genuchten-Mualem’s has a greater weightage, implying a superior fit overall. This is also supported by the sensitivity study done by Rahimi et al. [67], that Fredlund and Xing’s (1994) are shown to be able to curve fit the most accurately with a limited range of suction data available. The Van Genuchten model is widely used due to its clear physical interpretation of the parameters and effectiveness in fitting accuracy among a variety of soils with broad range of texture [48, 49, 68].

Therefore, variation between all the estimation models were studied independently from the soil database for the controlling factors: SWCC and equations as identified in this study. The parameter values presented in **Table 4** can serve as valuable initial references for researchers to model slopes of similar characteristics or references point for comparison. The data can be further improved with enhancement to the field monitoring equipment, to make it more precise in measuring the soil parameters. This will prevent the problem encountered when using the regression model, R^2^ as it will ensure the data collected to be more precise and accurate, resulting in an output that is as similar to a lab test as possible while still displaying the in-situ data with assurance. The investment in improving the precision of the equipment will boost the reliability and confidence of the actual result obtained. This will also reduce the number of assumptions, and the range of uncertainty boundaries required during calculation, allowing the model to be more realistic to the actual environment.

Field monitoring has shown great benefit to obtaining good sets of recorded data to be used to investigate the soil-water characteristic curve with real-time data on site. These are all special phenomena caused by rapid infiltration, that is, the change in soil suction or soil-water content lag behind on the other side in order to maintain the equilibrium of soil. Even for vertical infiltration at the same point, the changes in soil-water content and suction are not identical, which can be obtained by field monitoring. In contrast, the laboratory measurement can only be performed on a single soil sample and cannot understand the response of each soil horizon to a specific vertical infiltration event.

## Supporting information

S1 File S1 File for all the data and graph displayed. (XLSX)

## References

[1] Mohamed TA, Ali FH, Hashim S, Huat BBK. Relationship Between Shear Strength and Soil-water Characteristic Curve of an Unsaturated Granitic Residual Soil. American Journal of Environmental Sciences. 2006; 2(4): 142–145.

[2] Huat BBK, Ali FH, Sew GS. Tropical Residual Soils Engineering. In Taylor & Francis Group, London. 2004.

[3] Majid NA, Taha MR, Selamat SN. Historical landslide events in Malaysia 1993–2019. Indian Journal of Science and Technology. 2020; 13(33): 3387–3399.

[4] Taib AM, Taha MR, Rahman NA, Razuhanafi M, Yazid M. The Effect of Soil-Root Interaction by Vetiver Grass on Slope (January 2021). J. Eng. Sci. Technol. 2020; 15, 46–57.

[5] Howard TR, Baldwin JE, Donley HE. Landslides in Pacifica California, caused by the Storm. In: Landslides, Floods and Marine Effects of the Storm of January 3–5, 1982, in the San Francisco Bay Region, California In: SD Ellen, GF Wieckzoreck (eds):—U.S. Geological Survey Professional Paper 1434. 1988.

[6] Montrasio L, Valentino R. A model for triggering mechanisms of shallow landslides. Nat Haz Earth Sys Sci. 2008; 8, 1149– 1159. doi: 10.5194/nhess-8-1149-2008

[7] Ghani ANC, Taib AM, Hasbollah DZA. Effect of rainfall pattern on slope stability. In Geotech Sust Infrastruc Develop. 2020; 887–892.

[8] Sidle RC, Ochiai H. Landslides: Processes, Prediction, and Land Use, Water Res. Monogr. Ser. 18, 312 pp. AGU, Washington, D.C. 2006.

[9] Keefer DA, Larsen MC. Assessing landslide hazards, Science. 2007; 316, 1136–1138. 17525325 10.1126/science.1143308

[10] Godt JW, Baum RL, Lu N. Landsliding in partially saturated materials, Geophys. Res. Lett. 2009; 36, L02403. doi: 10.1029/2008GL035996

[11] Furuya G, Suemine A, Sassa K, Komatsubara T, Watanabe N, Marui H. Relationship between Groundwater Flow Estimated by Soil Temperature and Slope Failures Caused by Heavy Rainfall, Shikoku Island, Southwestern Japan. Eng. Geology. 2006; 85, 332–346. doi: 10.1016/j.enggeo.2006.03.002

[12] Cho S. Infiltration Analysis to Evaluate the Surficial Stability of Two-Layered Slopes Considering Rainfall Characteristics. Eng. Geology. 2009; 105, 32–43. doi: 10.1016/j.enggeo.2008.12.007

[13] Rahardjo H, Satyanaga A, Leong E. Effects of Flux Boundary Conditions on Pore-Water Pressure Distribution in Slope. Eng. Geology. 2012; 165, 133–142. doi: 10.1016/j.enggeo.2012.03.017

[14] Bordoni M, Meisina C, Valentino R, Lu N, Bittelli M, Chersich S. Hydrological Factors Affecting Rainfall-Induced Shallow Landslides: From the Field Monitoring to a Simplified Slope Stability Analysis. Eng. Geology. 2015; 193, 19–37. doi: 10.1016/j.enggeo.2015.04.006

[15] Lim TT, Rahardjo H, Chang MF, Fredlund DG. Effect of rainfall on matric suctions in a residual soil slope. Can Geotech J. 1996; 33:618–628. doi: 10.1139/t96-087

[16] Vanapalli SK, Fredlund DG, Pufahl DE, Clifton AW. Model for the prediction of shear strength with respect to soil suction. Can Geotech J. 1996; 33:379–392. doi: 10.1139/t96-060

[17] Godt JW, Baum RL, Savage WZ, Salciarini D, Schulz WH, Harp EL. Transient deterministic shallow landslide modelling: Requirements for susceptibility and hazard assessment in a GIS framework. Eng Geol. 2008; 102, 214–226. doi: 10.1016/j.enggeo.2008.03.019

[18] Baum RL, Godt JW, Savage WZ. Estimating the timing and location of shallow rainfallinduced landslides using a model for transient, unsaturated infiltration. Jour Geoph Res. 2010. doi: 10.1029/2009JF001321

[19] Lu N, Godt JW. Hillslope hydrology and stability. Cambridge University Press, Cambridge, U.K. 2013.

[20] Aung KK, Rahardjo H, Leong EC, Toll DG. Relationship between Porosimetry Measurement and Soil-Water Characteristic Curve for an Unsaturated Residual Soil. Geotechnical Geol. Eng. 2001; 19. 401–416. doi: 10.1007/978-94-015-9775-3_9

[21] Kristo C, Rahardjo H, Satyanaga A. Effect of variations in rainfall intensity on slope stability in Singapore Int. Soil Water Conserv. Res. 2017; 5(4): 258–264.

[22] Rahardjo H, Kim Y, Satyanaga A. Role of unsaturated soil mechanics in geotechnical engineering. International Journal of Geo-Engineering. 2019; 10(1). 10.1186/s40703-019-0104-8.

[23] Lu N. Generalized Soil-water Retention Equation for Adsorption and Capillarity. Journal of Geotechnical and Geoenvironmental Engineering. 2016; 142(10). 10.1061/(asce)gt.1943-5606.0001524.

[24] Fredlund DG. Relationship between the laboratory soil-water characteristic curves and field stress state. In Proceedings of the Asia-Pacific Conference on Unsaturated Soils, Quilin, China, 14–16 October. 2015.

[25] Zhang F. Unsaturated soil property functions for high volume change materials. Ph.D. dissertation, University of Alberta, Edmonton, Alta. 2016.

[26] Zhang LM, Chen Q. Predicting bimodal soil-water characteristic curves. Journal of Geotechnical and Geoenvironmental Engineering. 2005; 131(5): 666–670.

[27] Zhai Q, Rahardjo H, Satyanga A. Uncertainty in the estimation of hysteresis of soil-water characteristic curve. Environmental Geotechnics. 2017.

[28] Satyanaga A, Rahardjo H, Chai JH. Numerical Simulation of Capillary Barrier System under Rainfall Infiltration in Singapore. ISSMGE International Journal of Geoengineering Case Histories. 2019; 5(1): 43–54. 10.4417/ijgch-05-01-04.

[29] Buckingham E. Studies on the Movement of Soil Moisture. US Department of Agriculture, Bureau of Soils No. 38. 1907.

[30] Childs EC, Collis-George N. Soil geometry and soil–water equiliboria. Discussions Faraday Soc. 1948; 3, 78–85.

[31] Gardner WR. Some steady-state solutions of the unsaturated moisture flow equation with application to evaporation from a water table. Soil Science. 1958; 85(4): 228–232.

[32] Gardner WR. Soil suction and water movement. In Pore Pressure and Suction in Soils: Conference Organised by the British National Society of the International Society of Soil Mechanics and Foundation Engineering. Butterworths, London. 1961. p. 137–140.

[33] Fredlund DG, Xing A. Equations for the soil-water characteristic curve. Canadian Geotechnical Journal. 1994; 31(4): 521–532.

[34] Fredlund DG, Morgenstern NR. Stress State Variables for Unsaturated Soils. Journal of the Geotechnical Engineering Division. 1977; 103, 447–446.

[35] Dunnicliff J. Geotechnical Instrumentation for Monitoring Field Performance. Wiley, New York. 1993.

[36] Stähli M, Sättele M, Huggel C, McArdell BW, Lehmann P, Van Herwijnen A, et al. Review article: Monitoring and prediction in Early Warning Systems (EWS) for rapid mass movements. Natural Hazards and Earth System Sciences (NHESS). 2014; 2, 7149–7179.

[37] Zhang J, Jiao JJ, Yang J. In situ rainfall infiltration studies at a hillside in Hubei Province, China. Eng Geol. 2000; 57(1):31–38.

[38] Tsaparas I, Rahardjo H, Toll DG, Leong EC. Infiltration characteristics of two instrumented residual soil slopes. Canadian Geotechnical Journal. 2003; 40:1012–1032.

[39] Rahardjo H, Lee TT, Leong EC, Rezaur RB. Response of a residual soil slope to rainfall. Can Geotech J. 2005; 42:340–351.

[40] Trandafir AC, Sidle RC, Gomi T, Kamai T. Monitored and simulated variations in matric suction during rainfall in a residual soil slope. Environ Geol. 2008; 55:951–961.

[41] Harris SJ, Orense RP, Itoh K. Back analyses of rainfall-induced slope failure in Northland Allochthon formation. Landslides. 2012; 9(3): 349–356. doi: 10.1007/s10346-011-0309-1

[42] Fredlund DG, Rahardjo H. Soil mechanics for unsaturated soils. Wiley, New York. 1993.

[43] Hawke R, McConchie J. In situ measurement of soil moisture and pore-water pressures in an ‘incipient’ landslide: Lake Tutira, New Zealand. Journal of Environmental Management. 2011; 92(2):266–274. doi: 10.1016/j.jenvman.2009.05.035 19926207

[44] Smith JB, Godt JW, Baum RL, Coe JA, Burns WJ, Lu N, et al. Hydrologic monitoring of a landslide-prone hillslope in the Elliott State Forest, Southern Coast Range, Oregon, 2009–2012. U.S. Geological Survey Open-File Report, 2013–1283, U.S. Geological Survey, Reston, VA. 2014.

[45] Taib AM, Taha MR, Hasbollah DZA. Influence of Initial Conditions on Unsaturated Groundwater Flow Models. Int. J. Eng. Technol. 2019; 8, 34–40.

[46] Eyo EU, Ng’ambi S, Abbey SJ. An overview of soil–water characteristic curves of stabilised soils and their influential factors, Journal of King Saud University—Engineering Sciences. 2022; 34 (1): 31–4, ISSN 1018-3639. 10.1016/j.jksues.2020.07.013.

[47] Van Genuchten MT. A closed form equation for predicting the hydraulic conductivity of unsaturated soils. SoilSci Soc Am J. 1980; 44:892–898.

[48] Haverkamp R, Parlange JY. Predicting the water retention curve from particle-size distribution: 1. Sandy soils without organic matter. Soil Science. 1986; 142(6): 325–339.

[49] Šimůnek J, van Genuchten MTh, Šejna M. Development and applications of the HYDRUS and STANMOD software packages and related codes. Vadose Zone Journal. 2003; 2(4): 619–631.

[50] Leong EC, Rahardjo H. Review of Soil-Water Characteristic Curve Equations. Journal of Geotechnical and Geoenvironmental Engineering. 1997; 123(12): 1106–1117.

[51] Xing X, Liu Y, Ma X. A modified van-Genuchten model for soil-water retention modeling by considering plant additives. Archives of Agronomy and Soil Science. 2018; 65(4): 435–449. 10.1080/03650340.2018.1506583.

[52] Šimůnek J, van Genuchten MTh, Šejna M. Recent developments and applications of the HYDRUS computer software packages. Vadose Zone Journal. 2013; 12(3). doi: 10.2136/vzj2013.05.0099

[53] Brooks RH, Corey AT. Hydraulic properties of porous media. Colorado State University, Hydrology Paper. 1964; 3(27): 1–27.

[54] Shen J, Hu M, Wang X, Zhang C, Xu D. SWCC of Calcareous Silty Sand Under Different Fines Contents and dry Densities. Frontiers in Environmental Science. 2021; 9. 10.3389/fenvs.2021.682907.

[55] Aldaood A. Impact of fine materials on the saturated and unsaturated behavior of silty sand soil. Ain Shams Engineering Journal. 2020; 11(3): 717–725. doi: 10.1016/j.asej.2019.11.005

[56] Gallage CPK, Uchimura T. Effects of Dry Density and Grain Size Distribution on Soil-Water Characteristic Curves of Sandy Soils. Soils and Foundations. 2010; 50(1): 161–172. 10.3208/sandf.50.161.

[57] Agus R, Sidik F, Nizam NMS. Application of Gardner and van Genuchten models to predict the soil-water retention curve for tropical peat soil. Jurnal Teknologi. 2016; 78(3–3): 83–89.

[58] Ng’eno V, Omuto C, Mbuge D, Too V. Assessment of Super Absorbent Polymer (SAP) on Plant Available Water (PAW) in Dry Lands. Engineering. 2023; 15(02): 90–105. 10.4236/eng.2023.152008.

[59] Li X, Li D, Guo J, Luo X. An improved Gardner model for predicting soil-water retention characteristics of loess soils. Soil and Tillage Research. 2020; 199, 104592.

[60] Liu L, Lu Y, Fu Y, Horton R, Ren T. Estimating soil-water suction from texture, bulk density and electrical resistivity. Geoderma. 2022; 409, 115630. 10.1016/j.geoderma.2021.115630.

[61] BSI. BS 1377: 1990—Methods of Test for Soils for Civil Engineering Purposes. British Standards Institute, Milton Keynes. 1990.

[62] Zhang P, Chen G, Wu J, Wang C, Zheng S, Yu Y, et al. The Application and Improvement of Soil–Water Characteristic Curves through In Situ Monitoring Data in the Plains. Water. 2022; 14, 4012. 10.3390/w14244012

[63] Crawford MM, Bryson LS, Woolery EW, Wang Z. Long-term landslide monitoring using soil-water relationships and electrical data to estimate suction stress. Engineering Geology. 2019; 251, 146–157.

[64] Krause P, Boyle D, Bäse F. Comparison of different efficiency criteria for hydrological model assessment. Adv. Geosci. 2005; 5, 89–97. 10.5194/adgeo-5-89-2005.

[65] Legates DR, McCabe GJ. Evaluating the use of “goodness‐of‐fit” measures in hydrologic and hydroclimatic model validation. Water Resources Res. 1999; 35(1), 233–241. 10.1029/1998WR900018.

[66] Taib AM, Taha MR, Hasbollah DZA. Validation of Numerical Modelling Techniques in Unsaturated Slope Behaviour (Pengesahan Teknik Pemodelan Berangka Dalam Tingkah Laku Cerun Tak Tepu). Jurnal Kejuruteraan SI 1. 2018; (5): 29–35. doi: 10.17576/jkukm-2018-si1(1)-05

[67] Rahimi A, Rahardjo H, Leong EC. Effect of range of soil–water characteristic curve measurements on estimation of permeability function. Engineering Geology. 2015; 185, 96–104. 10.1016/j.enggeo.2014.11.017.

[68] Huang SY, Barbour SL, Fredlund DG. Development and verification of a coefficient of permeability function for a deformable unsaturated soil. Canadian Geotechnical Journal. 1998; 35 (3), 411, 1998.

